# Leucine‐rich repeat kinase 2 (LRRK2) inhibition upregulates microtubule‐associated protein 1B to ameliorate lysosomal dysfunction and parkinsonism

**DOI:** 10.1002/mco2.429

**Published:** 2023-11-20

**Authors:** Kang Chen, Fei Tang, Bin Du, Zhe‐Zhou Yue, Ling‐Ling Jiao, Xu‐Long Ding, Qing‐Zhang Tuo, Jie Meng, Si‐Yu He, Lunzhi Dai, Peng Lei, Xia‐Wei Wei

**Affiliations:** ^1^ Department of Neurology and State Key Laboratory of Biotherapy National Clinical Research Center for Geriatrics West China Hospital, Sichuan University, and Collaborative Center for Biotherapy Chengdu P. R. China; ^2^ Guizhou Yiluoqini Techno. Co., Ltd, Guizhou Shuanglong Airport Economic Zone Guiyang P. R. China

**Keywords:** A53T, LRRK2, MAP1B, MPTP, Parkinson's disease

## Abstract

Mutations in *LRRK*2 (encoding leucine‐rich repeat kinase 2 protein, LRRK2) are the most common genetic risk factors for Parkinson's disease (PD), and increased LRRK2 kinase activity was observed in sporadic PD. Therefore, inhibition of LRRK2 has been tested as a disease‐modifying therapeutic strategy using the LRRK2 mutant mice and sporadic PD. Here, we report a newly designed molecule, FL090, as a LRRK2 kinase inhibitor, verified in cell culture and animal models of PD. Using the 1‐methyl‐4‐phenyl‐1,2,3,6‐tetrahydropyridine mice and *SNCA* A53T transgenic mice, FL090 ameliorated motor dysfunctions, reduced LRRK2 kinase activity, and rescued loss in the dopaminergic neurons in the substantia nigra. Notably, by RNA‐Seq analysis, we identified microtubule‐associated protein 1 (MAP1B) as a crucial mediator of FL090's neuroprotective effects and found that MAP1B and LRRK2 co‐localize. Overexpression of MAP1B rescued 1‐methyl‐4‐phenylpyridinium induced cytotoxicity through rescuing the lysosomal function, and the protective effect of FL090 was lost in MAP1B knockout cells. Further studies may be focused on the in vivo mechanisms of MAP1B and microtubule function in PD. Collectively, these findings highlight the potential of FL090 as a therapeutic agent for sporadic PD and familial PD without *LRRK*2 mutations.

## Graphical abstract

Graphical Abstract: FL090 is a novel small‐molecule LRRK2 kinase inhibitor. FL090 reduces the level of phosphorylation of LRRK2, increases MAP1B expression, and prevents lysosomal defects in PD. Consequently, FL090 prevents dopaminergic neuronal damage and motor dysfunction in PD mice models. This action is through the elevation of MAP1B, as overexpression or pharmacological activation of MAP1B ameliorates PD toxicities.

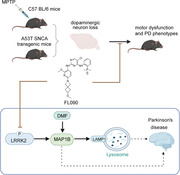

## 1 INTRODUCTION

Parkinson's disease (PD) is the second most common neurodegenerative disease, affecting about 2% of people over 65,^1^ and several genes have been identified to cause familial PD, including *SNCA* (encoding α‐synuclein), *PINK1* (encoding PTEN‐induced putative kinase 1, PINK1), *PRKN* (encoding Parkin), and *LRRK*2 (encoding leucine‐rich repeat kinase 2, LRRK2).^2^, ^3^ In particular, mutations in the *LRRK*2 gene represent the most prevalent genetic risk factors for PD,^4^ and the mutation of G2019S was the most common pathogenic variant in the LRRK2 protein, accounting for a high proportion (about 4%–5%) of familial PD cases and about 1% of sporadic PD cases.^5^ More importantly, noncoding variation at the *LRRK*2 locus is also associated with genome‐wide risk for PD,^6^ and reduced LRRK2 activity was reported in idiopathic PD.^7^, ^8^ Therefore, LRRK2 may be of significance in the pathogenesis of both familial and sporadic PD and the inhibition of LRRK2 kinase activity may represent a promising approach for disease modification.

LRRK2 is a serine/threonine kinase and structurally contains four functional domains, including the WD40 repeat domain, the leucine‐rich repeat domain (LRR), the guanosine triphosphate (GTP)‐binding regulatory domain, and the kinase domain.^9^ It was proposed to act on several cellular functions, including the regulation of cell signaling, modulation of cellular cytoskeleton dynamics, control of vesicle transport, and modulation of autophagy processes.^10^ Loss of LRRK2 has been identified to be associated with age‐dependent peripheral abnormalities,^11^, ^12^ and there are advantages to utilizing LRRK2 kinase inhibitors.^13^

Specific LRRK2 inhibitors have been developed, including DNL201 and DNL151 by Denali Therapeutics, PF‐06447475 by Pfizer, and BIIB094 by Biogen.^14^ DNL201 and DNL151 have the advantage of oral administration and have shown good safety and tolerability profile in early clinical trials.^4^ PF‐06447475 has been studied in early clinical trials and demonstrated safety, with potential improvement in non‐motor symptoms for *LRRK*2 mutation carriers.^14^ BIIB094, an antisense oligonucleotide developed to target LRRK2, has the potential to block abnormal aggregation of α‐syn and neuroinflammatory response^15^ and has undergone early clinical trials (NCT03976349). However, these LRRK2 inhibitors were mainly investigated in the context of *LRRK*2‐mutant PD, where increasing evidence also supports the role of LRRK2 in sporadic PD and familial PD without *LRRK*2 mutations.

In this study, our primary objectives were to investigate the therapeutic efficacy of FL090, a novel small‐molecule LRRK2 kinase inhibitor, in mitigating the motor disability and neurodegeneration associated with PD without *LRRK*2 mutation. To address this, we employed the 1‐methyl‐4‐phenyl‐1,2,3,6‐tetrahydropyridine (MPTP) intoxication model and *SNCA* A53T transgenic mice. These models are not directly associated with *LRRK*2 mutations, allowing us to assess the broader potential benefits of LRRK2 inhibitors in PD. We further conducted RNA‐seq analysis to investigate the mechanisms of action for FL090 and performed in vitro and in vivo experiments for validation. This line of experiments may provide further support for the use of LRRK2 inhibitors in PD, and further clues to the pathophysiological role of LRRK2 in PD.

## 2 RESULTS

### 2.1 FL090 reduced phosphorylation S935 of LRRK2 and prevented MPP^+^‐induced cytotoxicity and lysosomal damage

**FIGURE 1:**
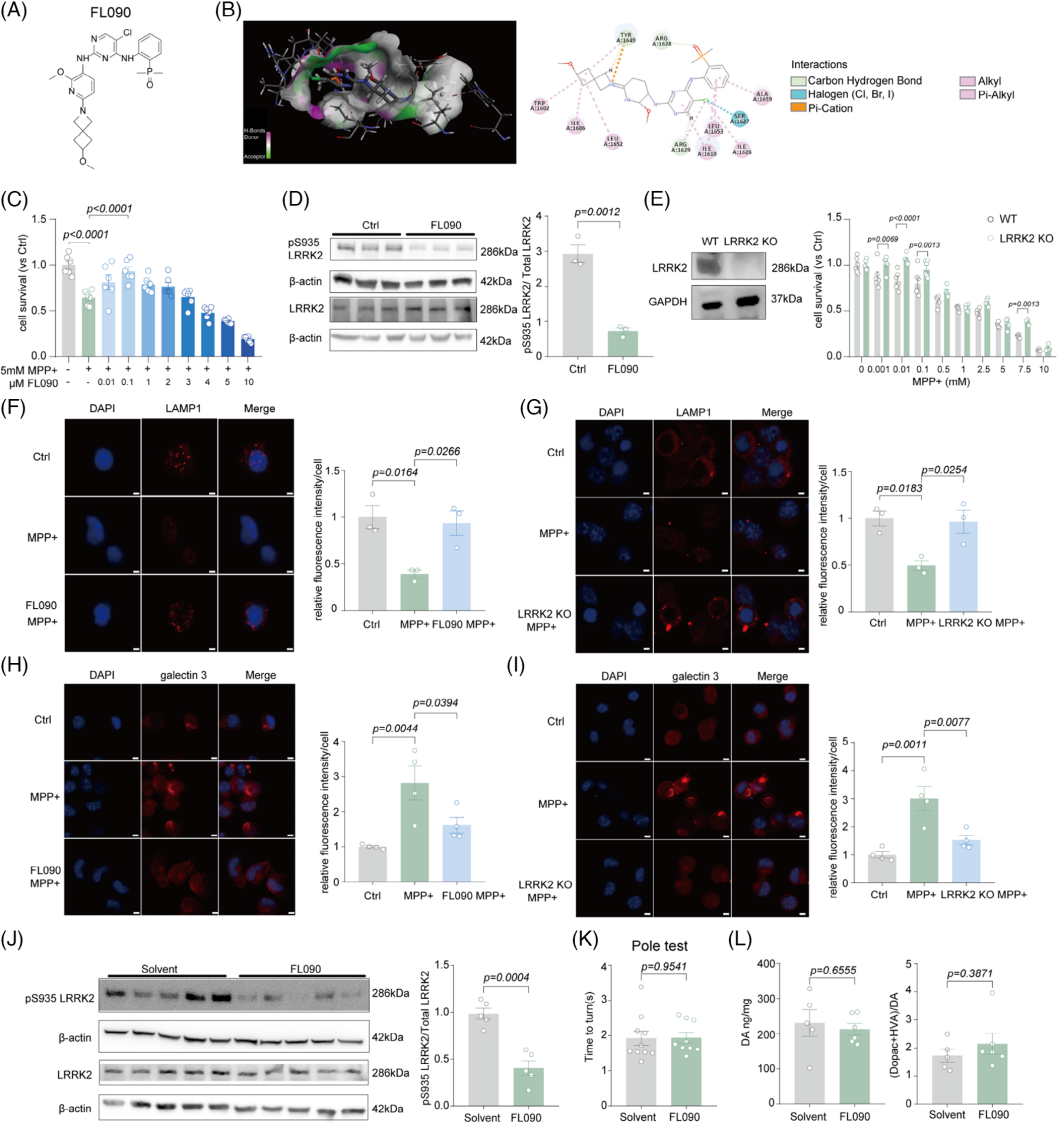
FL090 rescued 1‐methyl‐4‐phenylpyridinium (MPP^+^)‐induced cytotoxicity and lysosomal defects. (A) Molecular structure of FL090. (B) Molecular docking showing the interaction between FL090 and LRRK2. (C) Cell viability of N2a cells 24 h after MPP^+^ (5 mM) and FL090 of concentration gradient co‐treatment. Data are means ±  SEM, *n*  =  6 wells from one representative of three independent experiments. One‐way ANOVA with post hoc Tukey test was performed. (D) pS935 of LRRK2 and LRRK2 protein levels were examined in the N2a cells after 1 μM FL090 or DMSO treatment. Western blots were analyzed with ImageJ and normalized to β‐actin expression. Data are means ±  SEM, *n*  =  3. *t*‐Test was performed. (E) Cell viability in wild type (WT) cells and knockout LRRK2 cells 24 h after MPP^+^ of concentration gradient treatment. Data are means ±  SEM, *n*  =  6 wells from one representative of three independent experiments. Two‐way ANOVA with post hoc Sidak test was performed. (F) Immunofluorescence and quantification of LAMP1 in the cells after 5 mM MPP^+^ and 1 mand 1 fluorescence and quantbar = 3 μm. Data are means  ±  SEM, *n* = 3. One‐way ANOVA with post hoc Tukey test was performed. (G) Immunofluorescence and quantification of LAMP1 in the WT and LRRK2 KO cells after 5 mM MPP^+^ treatment. Scale bar = 3 μm. Data are means ±  SEM, *n* = 3. One‐way ANOVA with post hoc Tukey test was performed. (H) Immunofluorescence and quantification of Galectin 3 in the cells after 5 mM MPP^+^ and 1 μM FL090 co‐treatment. Scale bar = 3 μm. Data are means ±  SEM, *n* = 3. One‐way ANOVA with post hoc Tukey test was performed. (I) Immunofluorescence and quantification of Galectin 3 in the WT and LRRK2 KO cells after 5 mM MPP^+^ treatment. Scale bar = 3 μm. Data are means ±  SEM, *n* = 3. One‐way ANOVA with post hoc Tukey test was performed. (J) pS935 of LRRK2 and LRRK2 protein levels were examined in the substantia nigra (SN) of brain tissues isolated from mice with FL090 or solvent injection. Western blots were analyzed with ImageJ and normalized to β‐actin expression. Data are means ±  SEM, *n*  =  5. *t*‐Test was performed. (K) The pole test was performed after intragastric administration of FL090 for 18 days. The duration was recorded for the mouse to turn toward the ground. Data are means ±  SEM. Solvent, *n*  =  9; FL090, *n* = 10. t‐Test was performed. (L) The content of dopamine and its metabolites in the striatum was determined by liquid mass spectrometry. Data are means ±  SEM. Solvent, *n*  =  5; FL090, *n* = 6. t‐Test was performed. The *p* values are labeled in the histogram. Each point on the graph represents a sample.

FL090 (2‐((5‐chloro‐2‐((2‐methoxy‐6‐(6‐methoxy‐2‐azaspiro[3.3]heptan‐2‐yl) pyridin‐3‐yl) amino) pyrimidin‐4‐yl) amino) phenyl) dimethylphosphine oxide) may be a small‐molecule LRRK2 kinase inhibitor (Figure 1A). By simulation of Discovery Studio 2019 software, it is proposed that FL090 binds LRRK2 in the GTP‐binding regulatory domain (Figure 1B) which is related to its kinase activity.^16^ The cellular safety profile of FL090 was assessed using mouse neuroblastoma (N2a) cells and striatum‐derived human embryonic stem cell Huntington's disease Q7/7 (STHdh Q7/7) cells, and FL090 alone exhibited limited cytotoxicity (Figure S1A,B).

1‐methyl‐4‐phenylpyridinium (MPP^+^) induces cellular mitochondria damage by inhibiting respiration at complex I of the electron transport chain, which mimics pathological features of PD.^17^ FL090 effectively promoted cell survival after MPP^+^ treatment in N2a cells (Figure 1C), as well as in the STHdh Q7/7 cells (Figure S2). FL090 treatment also reduced the phosphorylation level of LRRK2 at the serine‐935 (pS935) site, as well as the phosphorylation level of RAB10 at the threonine at 73 (pT73), indicating that FL090 reduced the kinase activity of LRRK2 as designed (Figure 1D, Figure S3). We further examined if FL090 can specifically target LRRK2, by testing the expression of LRRK1, the human paralog of LRRK2, and RAB7, a substrate of LRRK1, and found no significant changes (Figure S3), indicating that the activity of LRRK1 was not affected by FL090. We then constructed LRRK2 knockout or knockdown cell lines by using CRISPR‐Cas9 system or *Lrrk*2 siRNA treatment (Figure 1E, Figure S4A), respectively, and genetic reduction of *Lrrk*2 prevented MPP^+^‐induced cytotoxicity similar to inhibition of LRRK2 activity by FL090 (Figure 1E, Figure S4B).

Lysosomal damage was commonly reported in PD associated with *LRRK*2 mutations,^18^ and we evaluated if MPP^+^‐induced lysosomal damage can be rescued by LRRK2 inhibition. Lysosomal‐associated membrane protein 1 (LAMP1/TMEM192) is a transmembrane protein primarily localized to lysosomes, and its expression serves as a marker for lysosomes.^19^, ^20^ We found that MPP^+^ significantly reduced the Lamp1 punctae, which was prevented by the co‐treatment of FL090 (Figure 1F), or knockout of LRRK2 (Figure 1G). Consistently, the lysosome acidification measured by lysosome trackers,^21^ essential for maintaining normal lysosomal functions,^22^ and the staining of galectin‐3 commonly used as an endocytosomal membrane damage marker^23^ indicate that lysosome function was impaired after MPP^+^ treatment, which can be prevented by FL090 treatment (Figure 1H, Figure S5A) or knockout of LRRK2 (Figure 1I, Figure S5B). Collectively, FL090 inhibited LRRK2 activity and prevented MPP^+^‐induced cytotoxicity and lysosomal damage in cells.

### 2.2 FL090 exhibited a safe profile ameliorated parkinsonism in the MPTP intoxication mice

To examine the safety profile of FL090 in the brain, we intragastrically administered FL090 (5 mg/kg, for 18 days, a dose we intended to treat the PD mice) to C57 BL/6 mice and observed significantly reduced LRRK2 kinase activity, evidenced by reduced phosphorylation at pS935 (Figure 1J). The prolonged treatment did not cause mortality, nor affect motor functions (Figure 1K, Figure S6A,B), nor the level of dopamine (DA) and its metabolites 3,4‐dihydroxybenzeneacetic acid (DOPAC) and homovanillic acid (HVA) in the striatum (Figure 1L), suggesting that FL090 has a favorable safety profile.

**FIGURE 2:**
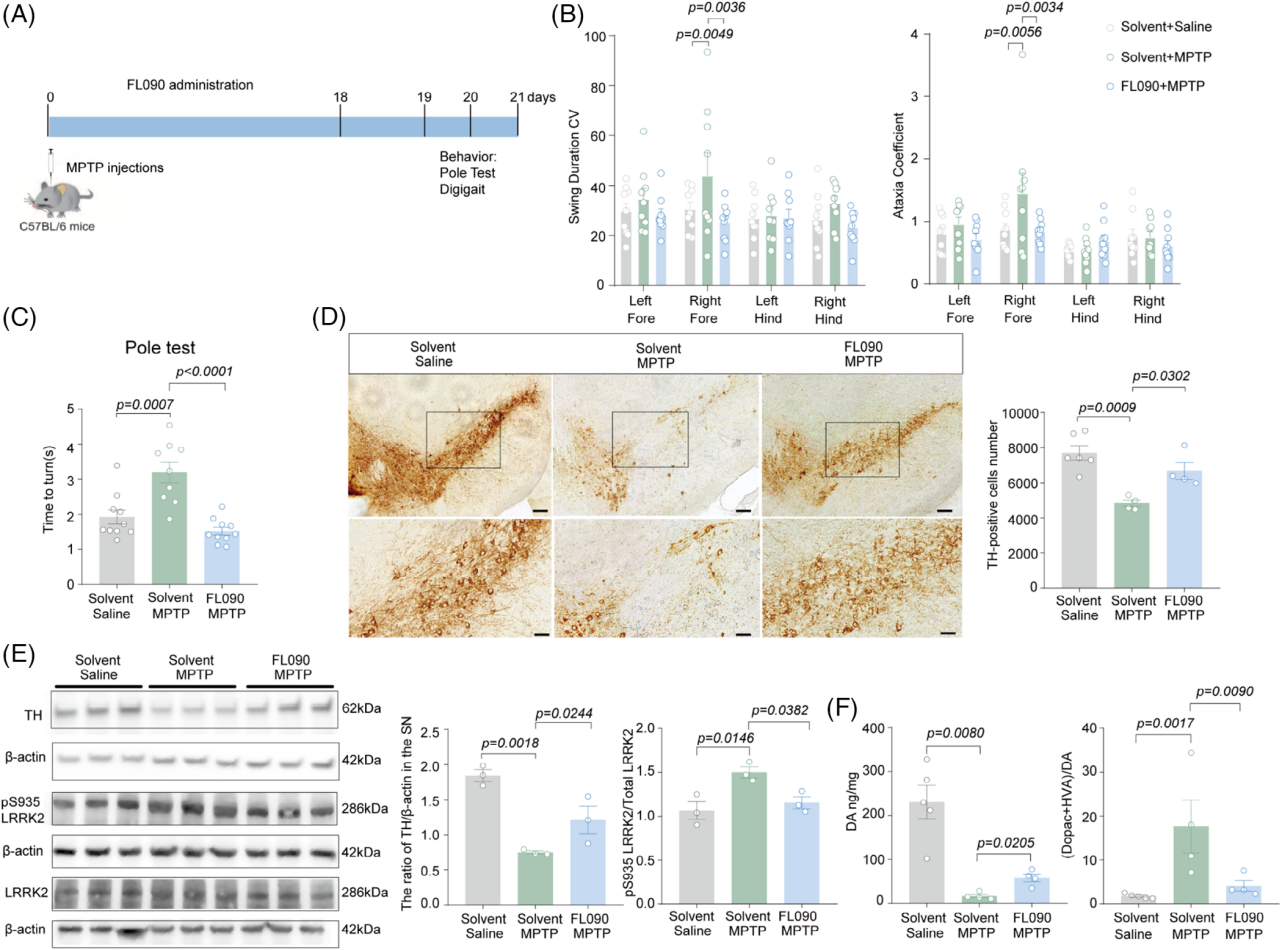
FL090 ameliorated motor dysfunctions and dopaminergic neuronal loss in the 1‐methyl‐4‐phenyl‐1,2,3,6‐tetrahydropyridine (MPTP) model. (A) Experimental scheme of model establishment, drug treatment, and behavior detection. (B) The swing duration of CV and ataxia coefficient were obtained from mice in the solvent + saline, solvent + MPTP, and FL090 + MPTP groups. The value on the *Y*‐axis represents the raw data exported from the DigiGait analysis system. Data are means  ±  SEM. Solvent + saline, *n*  =  10; solvent + MPTP, *n* = 9; FL090 + MPTP, *n* = 10. One‐way ANOVA with post hoc Tukey test was performed. (C) The pole test was performed after intragastric administration of FL090 for 18 days. The duration was recorded for the mouse to turn toward the ground. Data are means ±  SEM. *n*  =  9 animals per group. One‐way ANOVA with post hoc Tukey test was performed. (D) Immunohistochemistry staining and quantification of tyrosine hydroxylase (TH) in the solvent + saline, solvent + MPTP, and FL090 + MPTP groups. Data are means ±  SEM. Solvent + saline, *n*  =  6; solvent + MPTP, *n* = 4; FL090 + MPTP, *n* = 4. One‐way ANOVA with post hoc Tukey test was performed. The scale bars represent 200 and 400 μm, respectively. (E) Immunoblotting and quantification of TH protein, LRRK2, and pS935 LRRK2 levels in the substantia nigra (SN) of brain tissues isolated from the solvent + saline, solvent + MPTP, and FL090 + MPTP groups. Western blots were analyzed with ImageJ and normalized to β‐actin expression. Data are means ±  SEM, *n*  =  3. One‐way ANOVA with post hoc Tukey test was performed. (F) The content of dopamine and its metabolites in the striatum isolated from the solvent + saline, solvent + MPTP, and FL090 + MPTP groups were determined by liquid mass spectrometry. Data are means ±  SEM. Solvent + saline, *n*  =  5; solvent + MPTP, *n* = 4; FL090 + MPTP, *n* = 4. Brown–Forsythe and Welch ANOVA tests were performed. The *p* values are labeled in the histogram. Each point on the graph represents a sample.

To assess the therapeutic potential of FL090 in vivo, 8 weeks old C57 BL/6 mice were subjected to intraperitoneal (i.p.) injection of MPTP for 7 consecutive days, followed by intragastrical administration of FL090 (5 mg/kg) for 18 days (Figure 2A). FL090 prevented MPTP intoxication, evidenced by significantly decreased swing duration and ataxia coefficient of the right forelimbs in the DigiGait test (Figure 2B), reduced time‐to‐turn in the pole test (Figure 2C), and preserved dopaminergic neuron numbers in the substantia nigra (SN) (Figure 2D). The MPTP‐induced reduction in tyrosine hydroxylase (TH) expression was also prevented (Figure 2E), accompanied by reduced LRRK2 kinase activity (Figure 2E). Consistently, FL090 treatment also partially rescued the reduction of dopamine and changes in its metabolites (Figure 2F). Therefore, FL090 ameliorates motor dysfunctions in mice modeling sporadic PD (MPTP intoxication) with reduced LRRK2 kinase activity.

### 2.3 FL090 ameliorated parkinsonism in the *SNCA* A53T transgenic mice

The accumulation of α‐syn aggregates in specific brain regions is associated with dopaminergic neuron loss, motor deficits, and other pathological features observed in PD.^1^, ^24^, ^25^ *SNCA* A53T mutation was identified in human familial cases of PD,^26^ and the overexpression of this mutation in mice (*SNCA* A53T transgenic mice) leads to the formation of α‐syn aggregates and motor dysfunction.^27^ The mutation of *LRRK*2 was shown to promote α‐syn aggregation and propagation,^28^ and the inhibition of LRRK2 activity reduced α‐syn pathology.^8^, ^29^

**FIGURE 3:**
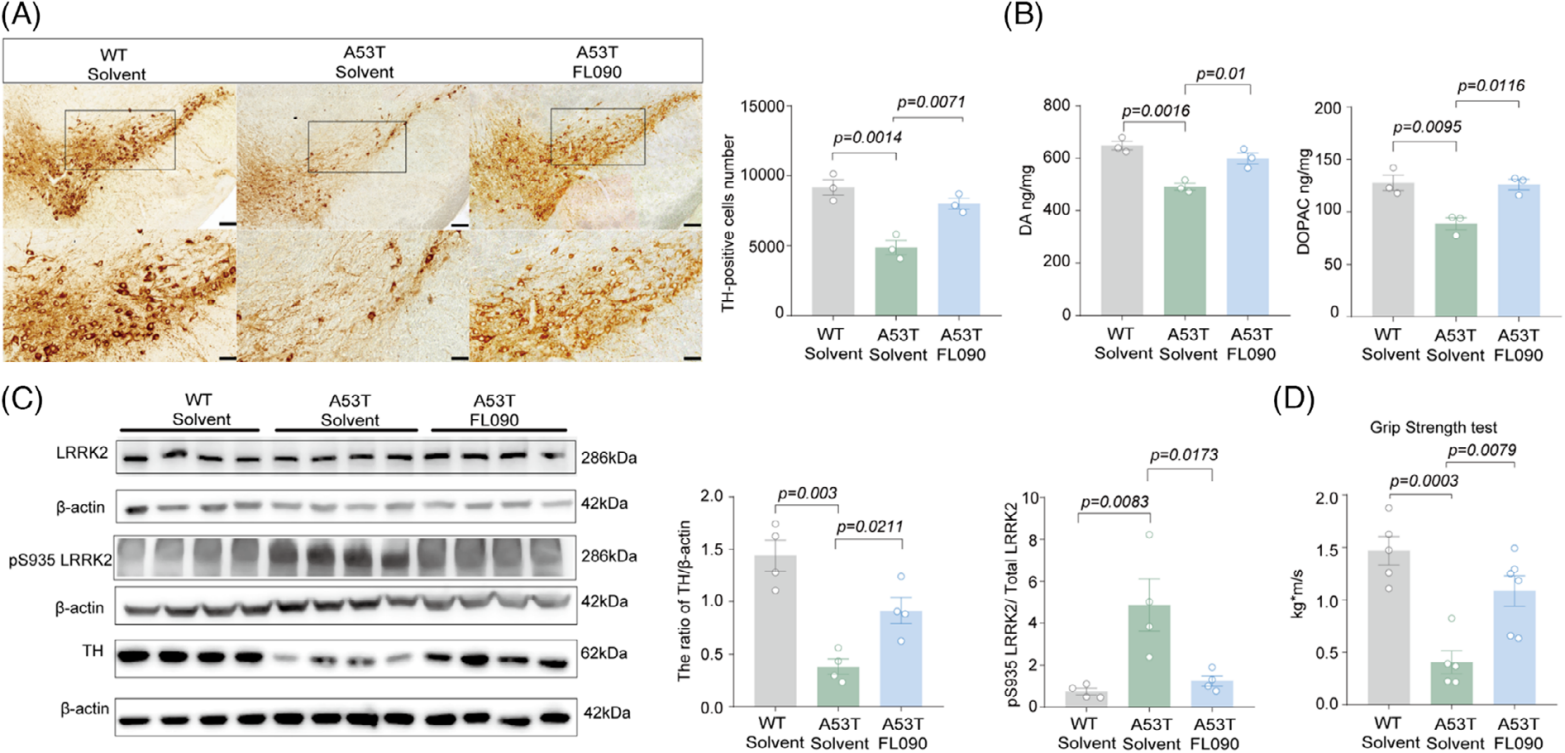
FL090 ameliorated motor dysfunctions and dopaminergic neuronal loss in *SNCA* A53T transgenic mice. (A) Immunohistochemistry staining and quantification of tyrosine hydroxylase (TH) in wild type (WT), *SNCA* A53T mice, and *SNCA* A53T mice with FL090 injection. The scale bars represent 200 and 400 μm, respectively. Data are means ±  SEM. *n*  =  3 animals per group. One‐way ANOVA with post hoc Tukey test was performed. (B) The content of dopamine and its metabolites in the striatum isolated from the WT, SNCA A53T mice, and SNCA A53T mice with FL090 injection were determined by liquid mass spectrometry. Data are means ±  SEM. *n*  =  3 animals per group. One‐way ANOVA with post hoc Tukey test was performed. (C) Immunoblotting and quantification of TH protein, LRRK2, and pS935 LRRK2 levels in the SN of brain tissues isolated from the WT, *SNCA* A53T mice, and *SNCA* A53T mice with FL090 injection. Western blots were analyzed with ImageJ and normalized to β‐actin expression. Data are means ±  SEM, *n*  =  4 animals per group. One‐way ANOVA with post hoc Tukey test was performed. (D) The grip strength test was performed after being injected with FL090 for 1 month by intragastric administration. The maximum value out of all trials was taken as a measure of grip strength. Data are means ±  SEM. WT, *n*  =  5; *SNCA* A53T, *n* = 5; FL090 + *SNCA* A53T, *n* = 6. One‐way ANOVA with post hoc Tukey test was performed. The *p* values are labeled in the histogram. Each point on the graph represents a sample.

FL090 was administered intragastrically into 9‐month‐old *SNCA* A53T transgenic mice for 30 days. In *SNCA* A53T mice, the number of dopaminergic neurons and the level of DA and DOPAC were significantly less compared with age‐matched wild type (WT) (Figure 3A,B), mimicking the phenotypes of parkinsonism as previously reported.^27^, ^30^, ^31^ FL090 treatment preserved the number of dopaminergic neurons in the SN and the reduced levels of DA, DOPAC, and TH expression (Figure 3A–C). As designed, the activated LRRK2 kinase in A53T mice was significantly suppressed by FL090 treatment (Figure 3C). We further evaluated the motor ability of the mice and found that the reduced grip strength of A53T mice was recovered by the FL090 treatment (Figure 3D). These results indicate that FL090 can be therapeutically beneficial in sporadic PD models and transgenic mice that are not directly linked with *LRRK2* mutation, further supporting the potential of FL090 for PD therapy. Furthermore, these results indicate that LRRK2 acts in the pathogenesis of sporadic PD.

### 2.4 Identifying key molecules mediating the neuroprotective effect of FL090

**FIGURE 4:**
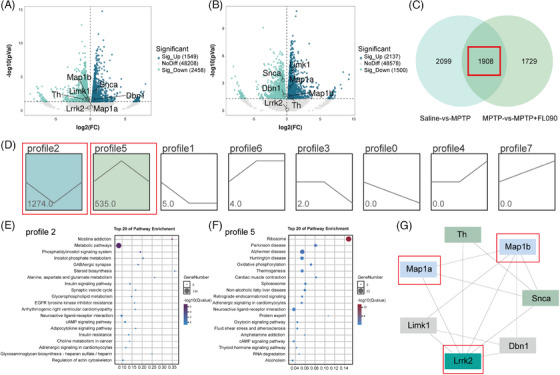
Screening key proteins involved in FL090 inhibition of LRRK2 activity. (A) Volcano plot of differentially expressed genes (DEGs) associated with Parkinson's disease (PD)‐related genes between the 1‐methyl‐4‐phenyl‐1,2,3,6‐tetrahydropyridine (MPTP) and the saline groups, with significant upregulations highlighted as blue dots and significant downregulations highlighted as green dots. (B) Volcano plot of DEGs associated with PD‐related genes between the FL090 and the MPTP groups, with significant upregulations highlighted as blue dots and significant downregulations highlighted as green dots. (C) Venn diagram depicting the overlap of DEGs between the MPTP versus saline groups and the FL090 versus MPTP groups. (D) The statistically significant profiles by trend analysis of genes at saline, MPTP, and FL090 group. The profiles that are colored represent statistically significant differences, *p* < 0.05. (E and F) Considering all pathways, the top 20 pathways were identified through an analysis of the Kyoto Encyclopedia of Genes and Genomes (KEGG) pathways of genes in profiles 2 (E) and 5 (F) during trend analysis. (G) Protein–protein interaction (PPI) network of LRRK2 with genes in profiles 2 and 5 during trend analysis.

To investigate the mechanism of the neuroprotective effects of FL090, we first analyzed the transcriptome data from the SN of the saline group, MPTP group, and MPTP + FL090 group. Bioinformatic analyses revealed significant changes in gene expression when comparing the saline group with the MPTP group (Figure 4A) or comparing the MPTP group with the MPTP + FL090 group (Figure 4B). Specifically, a total of 1908 shared differentially expressed genes (DEGs) were identified (Figure 4C), and trend analysis on RNA‐seq results over treatment groups indicated two significantly changed gene expression profiles (Figure 4D). Subsequently, the DEGs within these profiles were subjected to the Kyoto Encyclopedia of Genes and Genomes pathway enrichment analysis, where the PD pathway was enriched (Figure 4E). Protein–protein interaction analysis of LRRK2 using these DEGs identified MAP1B and MAP1A (Figure 4F), both of which belong to a protein family associated with microtubule functions.

### 2.5 MAP1B interacts with LRRK2 in PD models

**FIGURE 5:**
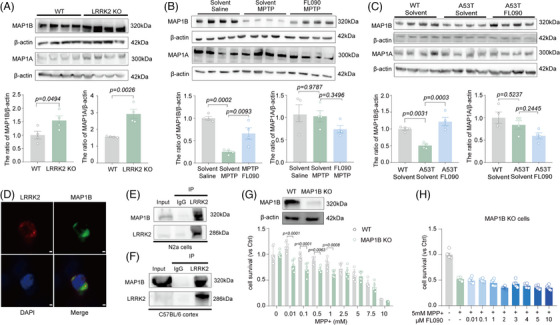
MAP1B plays an important role in the progress of FL090 rescued 1‐methyl‐4‐phenylpyridinium (MPP^+^)‐induced cytotoxicity. (A) Immunoblotting and quantification of MAP1B or MAP1A protein levels in the knockout of LRRK2 cells. Data are means ±  SEM. *n*  =  4. *t*‐Test was performed. (B) Immunoblotting and quantification of MAP1B or MAP1A protein levels in the substantia nigra (SN) of brain tissues isolated from the solvent + saline, solvent + 1‐methyl‐4‐phenyl‐1,2,3,6‐tetrahydropyridine (MPTP), and FL090 + MPTP groups. Data are means ±  SEM. *n*  =  4. One‐way ANOVA with post hoc Tukey test was performed. (C) Immunoblotting and quantification of MAP1B protein levels in the SN of brain tissues isolated from the wild type (WT), SNCA A53T mice, and SNCA A53T mice with FL090 injection. Data are means ±  SEM. *n*  =  4. One‐way ANOVA with post hoc Tukey test was performed. (D) Cellular co‐localization of endogenous LRRK2 and MAP1B. GFP‐MAP1B cells stained with LRRK2 (red). Scale bar = 2 μm. (E) Coimmunoprecipitation (Co‐IP) of endogenous MAP1B and LRRK2 in N2a cells. Rabbit IgG was used as a negative control. (F) Co‐IP of endogenous MAP1B and LRRK2 in C57BL/6 mouse cortical lysates. Rabbit IgG was used as a negative control. (G) Cell viability in WT cells and knockout MAP1B cells 24 h after MPP^+^ of concentration gradient treatment. Data are means  ±  SEM, *n*  =  6 wells from one representative of three independent experiments. Two‐way ANOVA with post hoc Sidak test was performed. (H) Cell viability of LRRK2 KO cells 24 h after MPP^+^ (5 mM) and FL090 of concentration gradient co‐treatment. Data are means ±  SEM, *n*  =  6 wells from one representative of three independent experiments. One‐way ANOVA with post hoc Tukey test was performed. The *p* values are labeled in the histogram. Each point on the graph represents a sample.

We first validated the results from the bioinformatics analysis and found that LRRK2 reduction by CRISPR‐Cas9 or siRNA markedly increased the level of MAP1B in N2a cells (Figure 5A, Figure S4A). MAP1B expression was also significantly reduced in the MPTP mice or A53T mice, whereas FL090 restored the reduced levels of MAP1B (Figure 5B,C). Spontaneously, MAP1A, which was also highlighted in the bioinformatic analysis, was unaltered in mice and was not affected by FL090 treatment (Figure 5B,C). To further determine if MAP1B was specifically affected by FL090, we examined another LRRK2 inhibitor (PF06447475) on the effect of MAP1B^32^ and found increased MAP1B after treatment with PF06447475 (Figure S7), similar to the effect of FL090. Therefore, MAP1B may be a co‐factor of LRRK2 action.

MAP1B is a crucial microtubule‐associated protein that plays a vital role in nerve cells by regulating microtubule assembly, stability, and neurodevelopment.^33^ It has been reported that the LRRK2 kinase can interact with the light chain portion of MAP1B (LC1) and MAP1B can rescue the toxicity of the *LRRK*2 mutant.^34^ We further observed that LRRK2 and MAP1B were subcellular co‐localized (Figure 5D), and MAP1B was able to be pulled down by LRRK2 in N2a cells (Figure 5E). Such binding between LRRK2 and MAP1B was further validated by coimmunoprecipitation (Co‐IP) in the cortex of WT mice (Figure 5F). We further found that MAP1B knockout in N2a cells worsened the MPP^+^ toxicities (Figure 5G), which could not be prevented by FL090 (Figures 1C and 5H) and MAP1B knockout did not affect LRRK2 expression (Figure S8A). Similar results were obtained in MAP1B knockdown cell lines by siRNA (Figure S8B,C).

**FIGURE 6:**
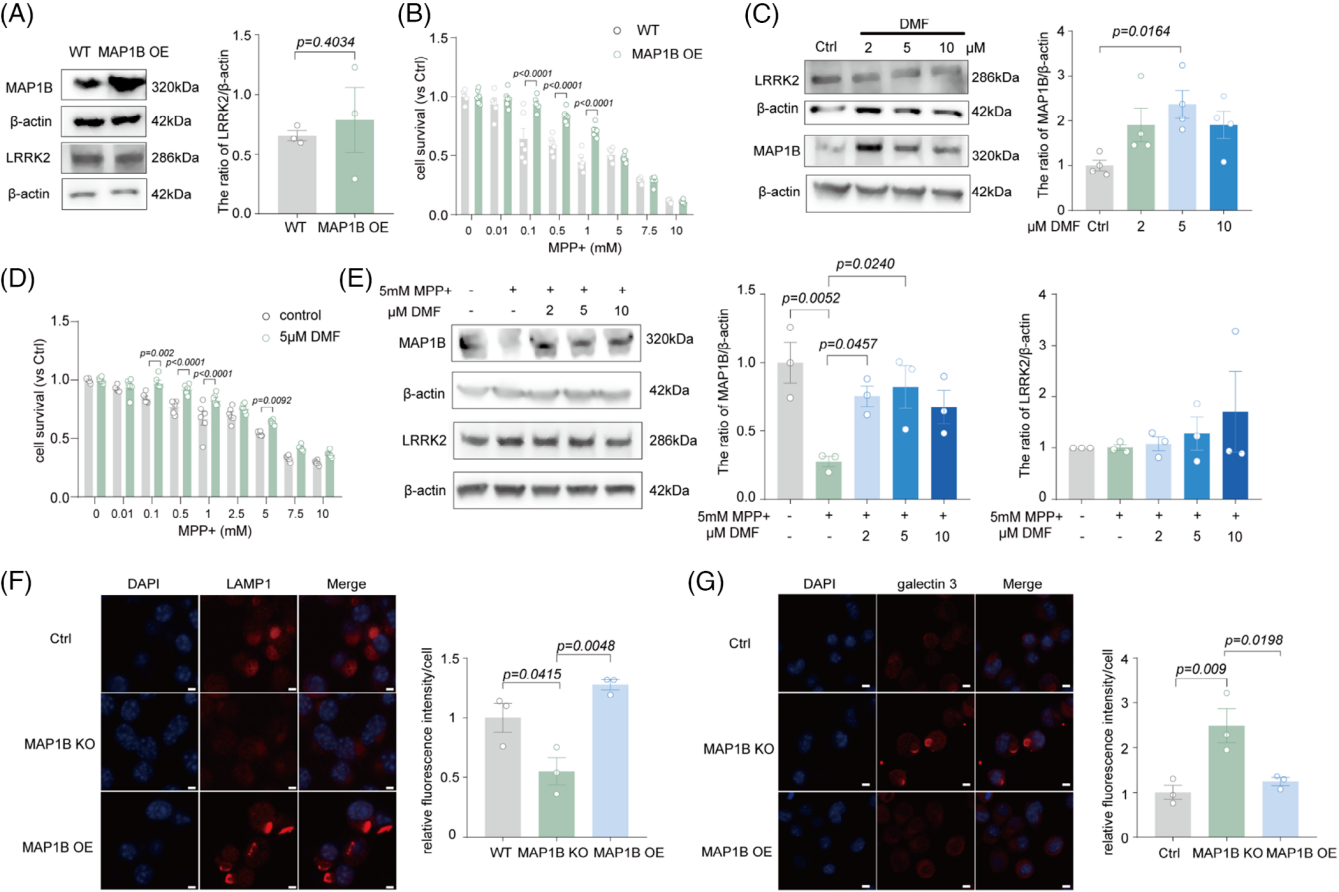
The interaction of LRRK2 and MAP1B. (A) Immunoblotting and quantification of LRRK2 protein levels in the GFP‐MAP1B cells. Data are means ±  SEM. *n*  =  3. *t*‐Test was performed. (B) Cell viability in wild type (WT) cells and overexpression MAP1B cells 24 h after 1‐methyl‐4‐phenylpyridinium (MPP^+^) of concentration gradient treatment. Data are means ±  SEM, *n*  =  6 wells from one representative of three independent experiments. Two‐way ANOVA with post hoc Sidak test was performed. (C) MAP1B protein level was examined in the N2a cells after dimethyl fumarate (DMF) of concentration gradient co‐treatment. Data are means ±  SEM, *n*  =  4. One‐way ANOVA with post hoc Tukey test was performed. (D) Cell viability of N2a cells 24 h after DMF (5 m fter DMF ^+^ of concentration gradient co‐treatment. Data are means ±  SEM, *n*  =  6 wells from one representative of three independent experiments. Two‐way ANOVA with post hoc Sidak test was performed. (E) MAP1B and LRRK2 protein levels were examined in the N2a cells after MPP^+^ (5 mM) and DMF of concentration gradient co‐treatment. Data are means ±  SEM, *n*  =  3. One‐way ANOVA with post hoc Tukey test was performed. (F) Immunofluorescence and quantification of LAMP1 in the MAP1B knockout and MAP1B overexpression cells. Scale bar = 3 μm. Data are means ±  SEM, *n* = 3. One‐way ANOVA with post hoc Tukey test was performed. (G) Immunofluorescence and quantification of Galectin 3 in the MAP1B knockout and MAP1B overexpression cells. Scale bar = 3 μm. Data are means ±  SEM, *n* = 3. One‐way ANOVA with post hoc Tukey test was performed. The *p* values are labeled in the histogram. Each point on the graph represents a sample.

Since LRRK2 inhibition rescued the MPP^+^ toxicity, we here investigated if MAP1B elevation has similar neuroprotective effects. Indeed, the overexpression of MAP1B rescued MPP^+^‐induced cytotoxicity (Figure 6A,B) without affecting the expression of LRRK2 (Figure 6A). Neuritic defects in PD result from reduced Nrf2 activity on antioxidant response elements at the *Map*1*b* locus.^33^ Dimethyl fumarate (DMF) is an Nrf2 activator, and it increased MAP1B expression by activating Nrf2.^33^ DMF treatment here also exhibited neuroprotection against MPP^+^ toxicity (Figure 6D). Moreover, it did not alter the expression of LRRK2 but significantly elevated the expression of MAP1B as predicted (Figure 6C,E). Lysosomal damages were also found in MAP1B knockout cells (Figure 6F,G, Figure S5C), similar to MPP+‐induced lysosomal damage, which was rescued by LRRK2 knockout (Figure 1G). These results collectively indicate that MAP1B can bind to LRRK2 and is a downstream factor of LRRK2 that is involved in MPTP toxicity.

## 3 DISCUSSION

We reported here that FL090, acting as an LRRK2 kinase inhibitor, ameliorated motor behavioral deficits and rescued the dopaminergic neuron loss in both intoxicated and α‐syn‐related parkinsonism rodent models. Treatment with FL090 also protected against MPP^+^‐induced cell death and lysosomal abnormalities, resembling the effects observed with LRRK2 knockout cells. RNA‐Seq analysis implicated MAP1B as a co‐factor of LRRK2, and we demonstrated that overexpression and pharmacological activation of MAP1B effectively rescued MPP^+^‐induced cytotoxicity. Notably, FL090 failed to restore cell survival in MAP1B knockout cells, indicating the critical role of MAP1B in mediating the protective effects of FL090.

As previously reported,^35^, ^36^, ^37^ sporadic PD accounts for approximately 85%–95% of all PD cases. It may result from a complex interplay of genetic susceptibility, environmental factors, age‐related changes, oxidative stress, mitochondrial dysfunction, protein misfolding and aggregation, and neuroinflammation.^38^, ^39^ Epidemiological studies across diverse populations have consistently demonstrated a relatively higher frequency (1%–2%) of mutations of *LRRK*2 in sporadic PD patients.^40^ Furthermore, genetic association studies have revealed an association between specific *LRRK*2 variants and the risk of sporadic PD.^41^, ^42^ However, the majority of existing LRRK2 inhibitors, including DNL201 and PF‐06447475, have predominantly been investigated in models of *LRRK*2 mutant PD models,^4^, ^32^ where the effect of LRRK2 may be different from that in sporadic PD. Therefore, we aim to address the role of LRRK2 inhibitors in parkinsonism models without *LRRK*2 mutations. We have calculated the binding domain of FL090 to LRRK2, which was similar to the previously reported LRRK2 inhibitors.^14^, ^43^ We further demonstrated here that the inhibition of LRRK2 by FL090 effectively prevented parkinsonism in toxin‐related and α‐syn‐mutant mice, highlighting its potential involvement in the pathogenesis of PD without *LRRK*2 mutations.

LRRK2 inhibitors have been shown with high inhibitory activity and selectivity against both WT and G2019S mutant *LRRK*2 mice, significantly inhibiting the Ser935 phosphorylation in the mouse brain.^14^ They also lead to the same Ser935 phosphorylation inhibition in peripheral tissues, raising concerns about potential toxicity.^14^ However, these concerns may be minor as no severe adverse events have been reported during clinical trials. We also found here that FL090 inhibited Ser935 phosphorylation of LRRK2 in the mouse brain at a lower dose, indicating its good safety profile. In addition, FL090 is a small compound, and its cLogP value meets the criteria for blood–brain barrier penetration. It also contains a substantial number of methyl and chlorine atoms, conferring it with favorable lipid solubility characteristics to cross the blood–brain barrier. However, we were focused on the changes in the brain after FL090 treatment and did not examine the potential abnormalities in the peripheral organs. Further research and clinical trials are still necessary to assess its safety and efficacy comprehensively.

Our study highlights a previously unrecognized role of MAP1B in PD. MAP1B is involved in the growth and guidance of axodendritic structures.^44^ There have been several reports on the presence of MAP1B within Lewy bodies extracted from postmortem cortical samples obtained from individuals diagnosed with Lewy body dementia,^45^, ^46^ and therefore, MAP1B may interact with α‐syn to form Lewy bodies.^46^ In our study, we found that MAP1B mediated the protective effect of FL090 on PD models, where FL090 was unable to prevent MPP^+^ toxicity in MAP1B knockout cells. The effect on MAP1B was not specifically induced by FL090, as another LRRK2 inhibitor also resulted in MAP1B elevation. Consistently, LRRK2 reduction resulted in an increased level of MAP1B. In contrast, the expression of MAP1B (either by overexpression or knockout) had a limited effect on the expression of LRRK2, indicating that MAP1B is a downstream factor of LRRK2. This interaction seems to be mediated by direct binding, evidenced by the co‐IP experiments. This line of evidence highlights a potential functional interplay between LRRK2 and MAP1B, which may be of importance in the pathogenesis of sporadic PD.

MAP1B belongs to the microtubule‐associated protein family, and members of this family, such as *MAPT* (tau protein),^47^ have also been implicated in PD.^48^, ^49^ Interestingly, an association between changes in tau pathology and *LRRK*2 mutations was identified in tauopathy.^50^ Individuals carrying *LRRK*2 mutations exhibit tau pathology to varying degrees.^51^ More recently, LRRK2 was highlighted as a genetic contributor to disease progression in the primary tauopathy progressive supranuclear palsy.^52^ These highlight the potential importance of LRRK2 in tauopathies, providing further support for the use of LRRK2 inhibitors. It is also noticed that tau was identified in the Lewy bodies in both PD and dementia with Lewy body patients,^53^ similar to the observations of MAP1B.^45^ Tau and MAP1B may share neuronal functions such as regulating microtubule dynamics,^54^, ^55^ where double knockout of both proteins was reported lethal.^54^ It is also reported that persistent tau reduction resulted in iron accumulation in dopaminergic neurons within the SN with age‐dependent loss of SN neurons and parkinsonism phenotypes,^56^, ^57^, ^58^, ^59^, ^60^ whereas the increased levels of MAP1B were discovered that may be a compensatory mechanism.^61^ Therefore, the microtubule‐associated protein family may be tractable targets for PD therapy.

In summary, our study demonstrates that LRRK2 kinase inhibitor effectively improves motor behavior deficits and protects dopaminergic neurons in *SNCA* A53T transgenic mice and sporadic PD models. We found that MAP1B interacts with LRRK2 and protects against Parkinsonism, both of which may be therapeutically targeted for the treatment of PD.

## 4 MATERIALS AND METHODS

### 4.1 Regents and antibodies

FL090 was obtained from Guizhou Yiluoqini Techno. Co., Ltd and dissolved in DMSO. DMSO (196055) was obtained from MP Biomedicals LLC. Anti‐GAPDH (G8795) antibodies, puromycin (P8833), polybrene (TR‐1003), phosphatase inhibitors II (P5726‐1ML) and III (P0044‐1ML), MTT (475989), and MPP^+^ (D048) were obtained from Sigma‐Aldrich. MPTP (s4732) was purchased from Selleck. Anti‐MAP1B (21633‐1‐AP) antibodies, anti‐RAB7A (55469‐1‐AP), anti‐Galectin‐3 antibody (60207‐1‐Ig), and anti‐LAMP1 (67300‐1‐Ig) antibodies were purchased from Proteintech. Isotype rabbit IgG (A7016), 4′,6‐diamidino‐2‐phenylindole (DAPI) (C1002), the bicinchoninic acid (BCA) Protein Assay Kit (P0009), the Cell lysis buffer for Western and immunoprecipitation (P0013B), Lyso‐Tracker Red (C1046), and the protease inhibitor phenylmethylsulfonyl fluoride (ST507) were purchased from Beyotime. Cy3 AffiniPure Goat Anti‐Mouse IgG (115‐165‐146) was purchased from Jackson. The transfection reagent Lipofectamine 3000, goat anti‐mouse antibody (31160), the transfection reagent Lipofectamine RNAiMAX, and HRP goat anti‐rabbit antibody (31460) were obtained from Thermo Fisher Scientific. Diaminobenzidine solution (ab64238), anti‐LRRK2 (ab133474), anti‐MAP1A (ab184350), anti‐pSer935 LRRK2 (ab133450), anti‐RAB10 (ab237703), anti‐pT73 RAB10 (ab230261), β‐actin (ab179467), and Goat Anti‐Rabbit IgG H&L Alexa Fluor 555 (ab150078) antibodies were purchased from Abcam. Anti‐TH (ab152) antibodies were purchased from Merck. Overall, 4% paraformaldehyde (PFA) (G1101) was purchased from Servicebio. Normal goat serum (NGS) (SL038) was obtained from Solarbio. Anti‐LRRK1 (A17768) antibody was purchased from Abclonal. PF06447475 (HY‐12477) was purchased from MCE. All the other chemicals and reagents were purchased from Sangon Biotech unless otherwise specified.

### 4.2 Animals

Adult male C57BL/6 mice (8 weeks old) were purchased from Chongqing Ensiweier Biotechnology Co. Ltd. Adult male *SNCA* A53T mice (8 weeks old) were purchased from Nanjing University Model Animal Research Center. All the mice were fed in a specific pathogen‐free facility under standard conditions of temperature (22 ± 2°C), humidity (45%–65%), and automatic light control (12 h light/12 h dark) at the State Key Laboratory of Biotherapy (Sichuan University). All experiments were approved by the Laboratory Animal Ethics Committee of Sichuan University (2021367A).

### 4.3 MPTP intoxication and drug treatment

Randomly assigned (by Excel ver. 2019) mice had received MPTP (30 mg/kg, i.p.) or equal amounts of 0.9% saline (i.p.) injection for 7 consecutive days.^17^ The DigiGait and pole tests were used to evaluate behavioral performances 19 days after the MPTP injection. For drug treatment, the Sham or MPTP mice received a daily injection of FL090 (5 mg/kg, intragastrical administration) or an equal amount of solvent (intragastrical administration) for 18 days.

### 4.4 Behavioral tests

#### 4.4.1 DigiGait test

The DigiGait imaging system (Mouse Specific, Inc.) was used for the recording and analysis of mouse movement, following previously established procedures.^17^ Each mouse was positioned on a transparent mechanical belt set to a consistent speed of 15 cm/s, which was selected to maintain stable walking; slower speeds induced exploratory walking, whereas faster speeds impeded walking. High‐speed cameras under the belt were employed to capture the walking behavior of the mice, and a 4s segment of steady walking was chosen for subsequent analysis using the DigiGait analysis system (Mouse Specifics, Inc.).

#### 4.4.2 Pole test

The pole test was performed as previously described.^58^ In the pole test, the apparatus consisted of a wooden pole and a ball on the top of the pole. The pole was 70 cm high, and 1 cm in diameter. During the pole test, the turning around time at the top of the pole and the total time it took for mice to get from the top to the bottom was measured. The apparatus was cleaned with 75% alcohol between each trial.

#### 4.4.3 Grip strength test

The grip strength test was employed to assess motor function in rodent models of CNS disorders.^62^ In this test, the paws of mice were positioned on a wire grid, which they would naturally grasp, whereas their tails were gently pulled backward. The maximum grip strength before release was recorded. The average performance of the animal for each session was calculated as the mean of the three trials. Typically, the testing period lasts 1 week.

### 4.5 Cell culture and viability assays

N2a (TCM29, National Collection of Authenticated Cell Cultures) and STHdh Q7/7 cells (From Dr. Boxun Lu, Fudan University) were cultured in Dulbecco's modified eagle medium (Gibco, Thermo Fisher Scientific) supplemented with 10% fetal bovine serum (Gibco, Thermo Fisher Scientific) and 1% penicillin–streptomycin. The culturing conditions involved maintaining the cells at 37°C in an environment with 5% CO_2_. Cells were seeded onto 96‐well plates and incubated for 24 h. Following this, the cells were treated with FL090, DMSO, or MPP^+^. Then, cell viability was evaluated using the MTT cytotoxicity assay kit (Sigma, M2003) as previously described.^13^

### 4.6 Establishment of MAP1B overexpressing cells

N2a cells were subjected to transfection using Lipofectamine 3000 (Invitrogen) following the manufacturer's instructions. Specifically, N2a cells were initially seeded in 6‐well plates and incubated for 24 h. When the cells reached a confluence of 70%, a transfection mixture was prepared. This mixture consisted of 125 μL of Opti‐MEM containing 2.5 μg of plasmids carrying GFP‐MAP1B (Addgene, 44396), along with 5 μL of P3000 Reagent and an additional 125 μL of Opti‐MEM containing 3.75 μL of Lipofectamine 3000 reagent. The cells were then incubated for 48 h. The cells were then harvested and subjected to protein analysis.

### 4.7 Establishment of LRRK2 knockout or MAP1B knockout cells using CRISPR‐Cas9 system

The sgRNAs were designed by online tools (http://crispr.mit.edu/ or https://crispr.cos.uni‐heidelberg.de/), and off‐target effects were assayed by http://asia.ensembl.org/. sgRNA was designed to specifically cut the first exon of the *Lrrk*2 or *Map*1*b* gene in the mouse genome (*Lrrk*2: Ensembl sequence ENSMUSEG00000036273 or *Map*1*b*: Ensembl sequence ENSMUSG00000052727). The *Lrrk*2‐sgRNA or *Map*1*b*‐sgRNA was cloned into a lentiCRISPRv2 vector, and the resulting construct was named sgRNA *Lrrk*2‐lentiCRISPRv2 or sgRNA *Map*1*b*‐lentiCRISPRv2. For transfection, sgRNA *Lrrk*2‐lentiCRISPRv2, sgRNA *Map*1*b*‐lentiCRISPRv2 or scramble DNA, and helper plasmids were transfected into HEK293FT cells by the calcium phosphate. The cells were seeded in 6‐well plates, and the amount of plasmid, psPAX2 (Addgene, 12260), and pMD2.G (Addgene, 12259) used for transfection in each well were 4, 2, and 1 μg, respectively. For infection, N2a cells were infected by treatment with viral supernatant and polybrene. After two rounds of infection, cells were stably selected and pooled with puromycin.

### 4.8 Establishment of LRRK2‐ or MAP1B‐knockdown cells by siRNA

Mouse *Lrrk*2 or *Map*1*b* siRNA and control siRNA (5′‐UUCUCCGAACGUGUCACGUTT‐3′) were obtained from GenePharma, and the target sequences for *Lrrk*2 and *Map*1*b* siRNA were as follows: siRNA *Map*1*b*‐1 (5′‐GCCCAAUGGUCAAGAAGUATT‐3′), siRNA *Map*1*b*‐2 (5′‐GGAGCUCAUUGAAGAUGAATT‐3′), siRNA *Map*1*b*‐3(5′‐GACACCCAAUGAGAUUAAATT‐3′), siRNA *Lrrk*2‐1 (5′‐GGACAUUGCAAAGCUUAAUTT‐3′), and siRNA *Lrrk*2‐2 (5′‐GCUGCAGGAUGGGAAUAAATT‐3′). The N2a cells were then transfected with siRNA using Lipofectamine RNAiMAX Transfection Reagent according to the manufacturer's instructions to obtain LRRK2‐ and MAP1B‐knockdown cells. Specifically, when the cells reached 70% confluence, 150 μL of Opti‐MEM containing 1.5 μL of 20 μM siRNA, and 150 μL of Opti‐MEM containing 9 μL of Lipofectamine RNAiMAX were mixed and added, and the cells were incubated for 48 h. Then the cells were harvested and analyzed for LRRK2 and MAP1B expression by Western blot.

### 4.9 Molecular docking

The crystal structures of the ligand binding domain from human LRRK2 (PDB number: 7LHW) were obtained from the RCSB PDB database (https://www.rcsb.org/). The chemical structures of FL090 were imported into Discovery Studio 2019 software (Biovia) and subjected to optimization processes. The target protein structure was also imported into Discovery Studio 2019 software, and several protein modifications were performed, including the removal of water and small ligand molecules, the addition of hydrogen atoms, cleaning the protein to eliminate excess protein structure, defining the protein as the receptor, and generating a docking site. Molecular docking with the target small molecule was carried out using the LibDock module.^63^, ^64^ The LibDock score was used to assess the affinity of the molecular conformation.

### 4.10 Western blot

Cells were lysed using RIPA lysis buffer, which consisted of 50 mM Tris–HCl (pH = 7.6), 150 mM NaCl, 1% (v/v) Triton X‐100, phenylmethylsulfonyl fluoride (1:100) as a protease inhibitor, and phosphatase inhibitors II and III (1:1000). This lysis process was conducted on ice for 25 min, followed by centrifugation at 13,000 × *g* for 25 min at 4°C to collect the supernatants for analysis.

For mice, they were deeply anesthetized and transcardially perfused with 0.01 M PBS. Subsequently, the striatum and SN regions of the brain were carefully dissected on ice. The tissue was then homogenized using RIPA lysis buffer on ice for 30 min, which helps to break down the cells and release their contents. After homogenization, the samples were subjected to centrifugation at 13,000 × *g* for 25 min at 4°C, and the supernatants were collected for Western blot.

To determine protein concentration, a BCA protein assay kit was employed. Equal amounts of protein from each sample were mixed with loading buffer, denatured at 95°C for 10 min and then separated using 4%–20% SDS polyacrylamide gels. The proteins within the gels were subsequently transferred onto PVDF membranes (Millipore). The membranes were blocked using 5% nonfat milk and then probed with suitable primary antibodies followed by secondary antibodies conjugated with IgG‐HRP (1:10000). An enhanced chemiluminescence detection system (Thermo Scientific) was used for development, and a ChemiDoc XRS^+^ system (Bio‐Rad) was used for visualization. Immunoreactive signals were quantified using ImageJ software (version 1.49, NIH). The primary antibodies used in this study targeted TH (1:5000), LRRK2 (1:1000), MAP1B (1:1000), MAP1A (1:1000), pSer935 LRRK2 (1:1000), LRRK1 (1:1000), RAB7 (1:1000), RAB10 (1:1000), pT73 RAB10 (1:1000), GAPDH (1:5000), and β‐actin (1:5000). Uncropped images of Western blots can be found in Figure S9.

### 4.11 Coimmunoprecipitation

Anti‐LRRK2 (1:50) and isotype rabbit IgG (1:100) were conjugated to magnetic beads to form antibody‐carrier complexes. The isotype rabbit IgG (1:100) alone was used as a negative control. These complexes were then incubated with the mouse cortical lysates to allow specific binding of the antibodies to the target protein, resulting in the formation of protein complexes. Following a series of washes to remove nonspecifically bound proteins and impurities, the immunocomplexes were denatured using SDS‐PAGE sample buffer or heat, causing the dissociation of the antibody‐protein interactions. Subsequently, the samples were subjected to SDS‐PAGE separation, followed by immunoblotting using specific antibodies to detect the presence of the target protein and its interacting partners.

### 4.12 TH staining

Mice were subjected to transcardial perfusion with PBS, followed by fixation with 4% PFA. Subsequently, the brain was carefully extracted and immersed in 4% PFA for overnight fixation. After fixation, the brain tissue underwent dehydration by immersion in 30% sucrose at 4°C until the brain sank to the bottom, as described previously.^65^ The SN was then sliced into 30 μm sections using a cryostat microtome (CM1860, Leica). The slices were blocked in 6% NGS for 2 h at room temperature. Following blocking, the sections were incubated overnight at 4°C with a primary antibody directed against TH (1:1000). After primary antibody incubation, the slices were treated with secondary antibodies conjugated with IgG‐HRP (1:500) for 2 h at room temperature. Afterward, the slices were followed by exposure to diaminobenzidine solution (1:50) for 30 s and then washing for 5 min to complete the staining process.^66^

### 4.13 Immunocytochemistry staining

Cells were fixed with 4% PFA at room temperature for 10 min. Subsequently, the fixed cells were washed three times for 5 min. Then the cells were blocked for 2 h at room temperature with 6% NGS and incubated overnight at 4°C with primary antibodies. The primary antibodies utilized in this study targeted LAMP1 (1:1000), Galectin 3 (1:1000), and LRRK2 (1:1000). In the subsequent day, cells were washed three times for 10 min and incubated with a secondary antibody, specifically the Cy3 AffiniPure Goat Anti‐Mouse IgG (1:500), for 2 h at room temperature. After another three washes, the cell nuclei were stained with DAPI for 10 min. To visualize the results, images were captured using a fluorescent microscope (Zeiss, LSM 880). At least three nonoverlapping images were analyzed using ImageJ (version 1.49, NIH) in a double‐blinded manner.

### 4.14 Lyso‐Tracker Red fluorescent staining

A small amount of Lyso‐Tracker Red (1:15000) was added to the cell culture medium. The cell culture medium was removed and added the pre‐warmed Lyso‐Tracker Red staining solution at 37°C. The cells were incubated together with the staining solution at 37°C for 5–60 min. After incubation, the Lyso‐Tracker Red staining solution was removed and replaced with a fresh cell culture medium. Then lysosomes can be typically observed using fluorescence microscopy or laser confocal microscopy. Bright strong fluorescent staining of lysosomes can be observed.

### 4.15 Stereological estimation of TH‐positive neuron number

TH‐positive neurons in the SNpc were quantified using an automated stereological analysis approach, as previously described.^56^ In summary, this process involved the implementation of the Stereo Investigator software package (version 2017, MicroBrightField Inc.). The SNpc was outlined at a 10× objective (numerical aperture 0.25), and a sampling grid was placed over the area of interest. TH immunopositivity cells were then observed at high magnification (40× objective with numerical aperture 0.8). Only TH immunoactivity‐positive cells were included in the count.

### 4.16 Dopamine and its metabolite measurements

The striatum was homogenized using Optima LC/MS Grade water (Fisher Chemical) and then mixed with Optima LC/MS Grade acetonitrile (Fisher Chemical). The resulting mixture was subsequently centrifuged at 14,000 rpm for 15 min at 4°C. After centrifugation, the supernatants were collected, filtered, and sequentially evaporated. The samples were redissolved in acetonitrile and then introduced into a chromatographic column (ACQUITY UPLC HSS T3 1.8 m), using the LC–MS/MS system (AB SCIEX QTRAP 5500) to detect DA and its metabolites, DOPAC and HVA.^67^

### 4.17 RNA‐Seq and data analysis

Total RNA isolated from the SN of the brain was extracted using Trizol reagent (Invitrogen) following the manufacturer's guideline. Quantity and purity were analyzed of Bioanalyzer 2100 and RNA 6000 Nano LabChip Kit (Agilent) with RIN score >7.0 as the cutoff. Approximately 1 μg of total RNA representing a specific adipose type was subjected to isolate Poly (A) mRNA with poly‐T oligo‐attached magnetic beads (Invitrogen). Following purification, the mRNA is fragmented into ∼600 nt long oligonucleotides using divalent cations under elevated temperature. The cleaved RNA fragments were reverse‐transcribed to create the final cDNA library by the protocol for the mRNA Seq sample preparation kit (Illumina) by the dUTP method, and the average insert size for the paired‐end libraries was 300 bp (±50 bp). Subsequent paired‐end 2 × 150 bp (PE150) sequencing was performed on an Illumina Novaseq 6000 platform at the Novogene following the vendor's recommended protocol. There were three replicates per experimental condition. All sequence reads were aligned to the *Mus musculus* GRCm38 reference genome using STAR (version 2.7.1a). Subsequently, we quantified the read counts for each gene with feature count in the Subread bundle according to Gencode (version. M2) annotation. Differential expression (DE) analysis was performed using the DESeq2 package in R, version 3.5.2. Only those genes with an average normalized expression value ≥1 were included in our DE analysis.

### 4.18 Statistical analysis

Data from independent experiments were expressed as the mean ± SEM, and an unpaired Student's *t*‐test, one‐way ANOVA followed by Dunnett's post hoc test, or two‐way ANOVA followed by Tukey's post hoc test were performed for statistical analysis by using GraphPad Prism 8 (GraphPad). For all analyses, statistical significance was defined as *p* < 0.05.

## AUTHOR CONTRIBUTIONS

Peng Lei and Xia‐Wei Wei conceived, raised funds for, and supervised the overall project. Kang Chen, Fei Tang, and Ling‐ling Jiao carried out the animal experiments. Kang Chen, Bin Du, and Zhe‐zhou Yue performed cell culture experiments. Bin Du and Xu‐long Ding analyzed the mouse transcriptome data. Si‐yu He performed dopamine and DOPAC measurements with the supervision of Lunzhi Dai. Qing‐zhang Tuo and Jie Meng critically revised the work. Kang Chen, Xia‐Wei Wei, and Peng Lei integrated the data and wrote the drafts of the manuscript. All authors have edited, read, and approved the final manuscript.

## CONFLICT OF INTEREST STATEMENT

X.W. and Z.Y. are shareholders of Guizhou Yiluoqini Techno. Co., Ltd. All other authors declare no conflict of interest.

## ETHICS STATEMENT

All mouse experiments were approved by the Laboratory Animal Ethics Committee of Sichuan University (2021367A).

## Supporting information

Supporting information

## Data Availability

RNA‐seq datasets (syn52249105) are available at (https://www.synapse.org/#!Synapse:syn52249098). To access the data, a data use agreement is needed. The data that support the findings of this study are available from the corresponding author upon reasonable request.
