# Supplementary material for: Leucine‐rich repeat kinase 2 (LRRK2) inhibition upregulates microtubule‐associated protein 1B to ameliorate lysosomal dysfunction and parkinsonism
Source: MedComm (2020). 2023 Nov 20;4(6):e429. doi: 10.1002/mco2.429 (PMC10661827; doi:10.1002/mco2.429)
Supplement: Supplementary file 1 — Supporting information [file MCO2-4-e429-s001.docx]

**Supplementary information**

**Leucine-Rich Repeat Kinase 2 Inhibition Upregulates Microtubule-Associated Protein 1B to Ameliorate Lysosomal Dysfunction and Parkinsonism**

Kang Chen^1#^, Fei Tang^1#^, Bin Du^1#^, Zhe-zhou Yue^2^, Ling-ling Jiao^1^, Xu-long Ding^1^, Qing-zhang Tuo^1^, Jie Meng^1^, Si-yu He^1^, Lunzhi Dai^1^, Peng Lei^1^*, Xia-Wei Wei^1,2^*

^1^ Department of Neurology and State Key Laboratory of Biotherapy, West China Hospital, Sichuan University, and Collaborative Center for Biotherapy, Chengdu, 610041, P.R. China

^2^ Guizhou Yiluoqini Techno. Co., Ltd, Guizhou Shuanglong Airport Economic Zone, Guiyang, 550005, P.R. China

^#^Kang Chen, Fei Tang, and Bin Du contributed equally to this work.

*Correspondence to

Xia-Wei Wei, E-mail: xiaweiwei@scu.edu.cn;

Peng Lei, E-mail: peng.lei@scu.edu.cn;


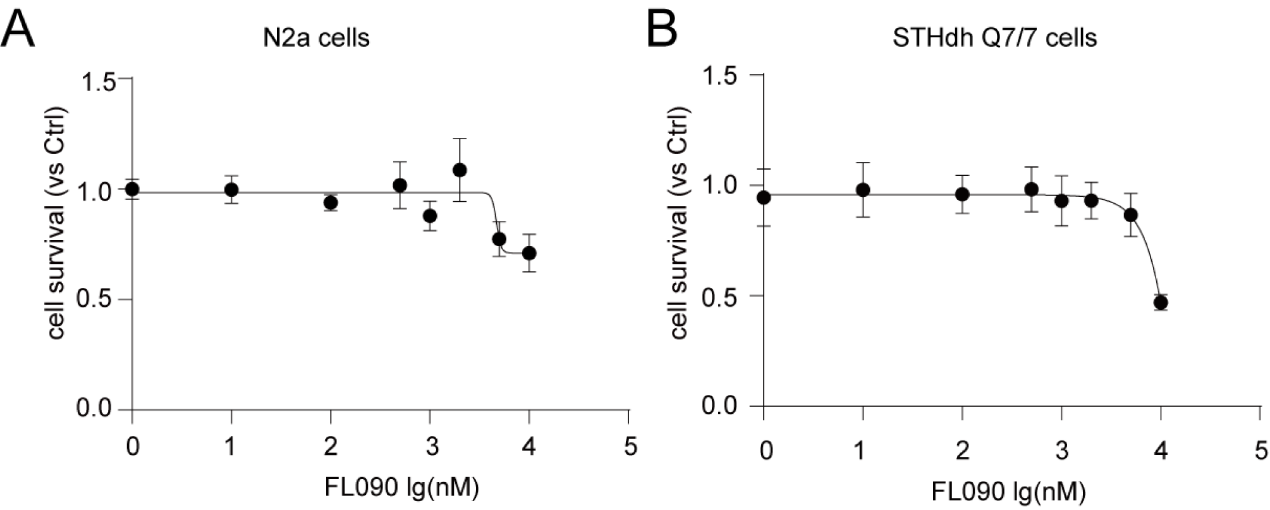


Supplementary Figure 1. **A-B** The cytotoxicity of FL090 in N2a, and STHdh Q7/7 cells. Data are means ± SEM, n = 6 wells from one representative of 3 independent experiments.


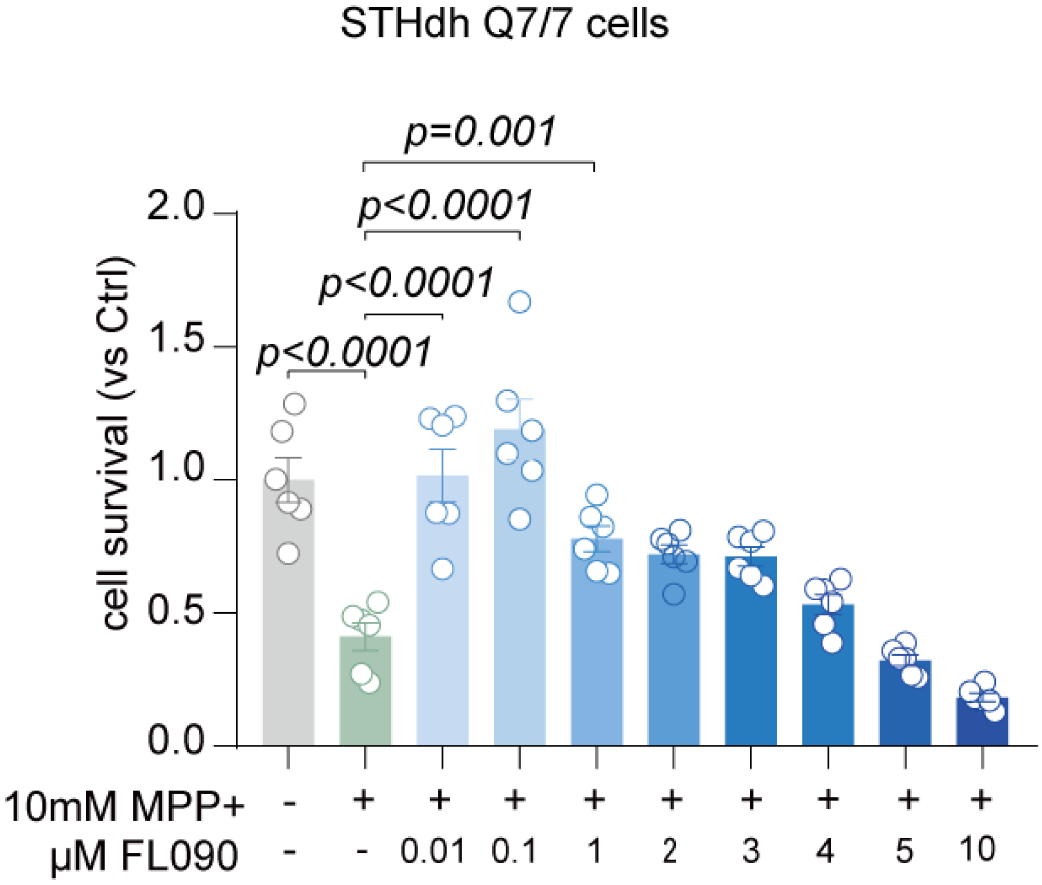


Supplementary Figure 2. Cell viability of STHdh Q7/7 cells 24 h after MPP+ (10mM) and FL090 of concentration gradient co-treatment. Data are means ± SEM, n = 6 wells from one representative of 3 independent experiments. One-way ANOVA with post-hoc Tukey test was performed. The p values lower than 0.05 are labeled in the histogram; otherwise, the p values are not displayed. Each point on the graph represents a sample.


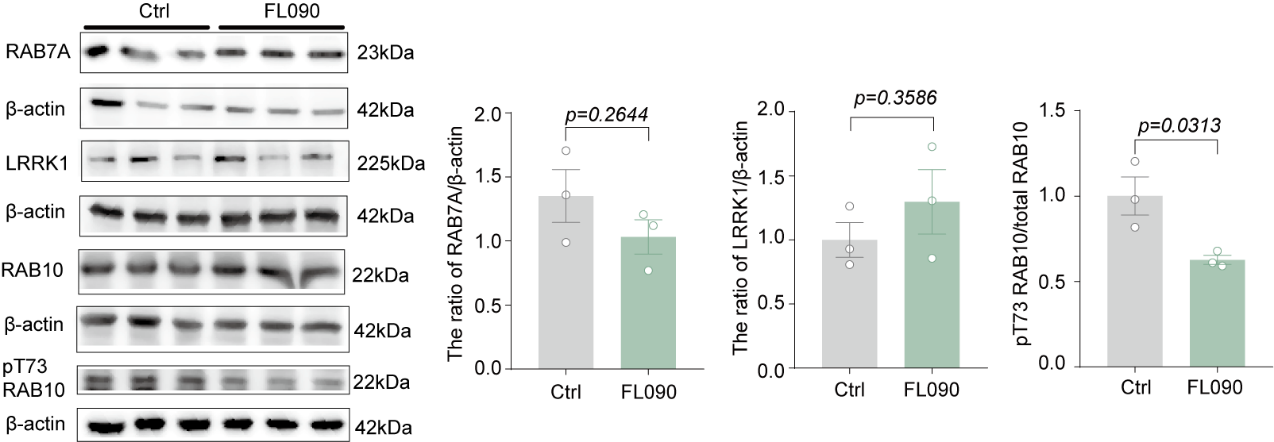
 Supplementary Figure 3. RAB7A, LRRK1, RAB10 and pT73 RAB10 protein levels were examined in the N2a cells after 1 μM FL090 or DMSO treatment. Western blots were analyzed with Image J and normalized to β-actin expression. Data are means ± SEM, n = 3. t-test was performed. The p value as indicated in the figure. Each point on the graph represents a sample.


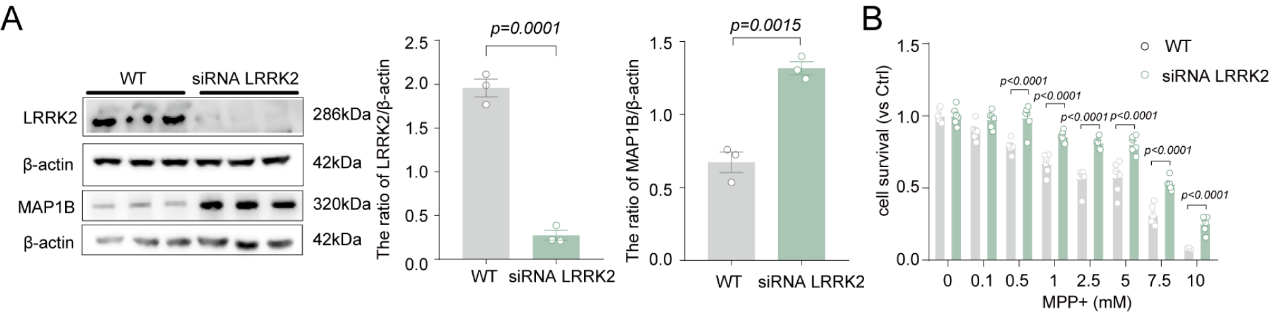


Supplementary Figure 4. Knockdown of LRRK2 ameliorated cell death induced by MPP+. **A** MAP1B and LRRK2 protein level were examined in the knockdown of LRRK2 cells. Western blots were analyzed with Image J and normalized to β-actin expression. Data are means ± SEM, n = 3. t-test was performed. **B** Cell viability in WT cells and Knockdown LRRK2 cells 24 h after MPP+ of concentration gradient treatment. Data are means ± SEM, n = 6 wells from one representative of 3 independent experiments. Two-way ANOVA with post-hoc Sidak test was performed. The p value as indicated in the figure. Each point on the graph represents a sample.


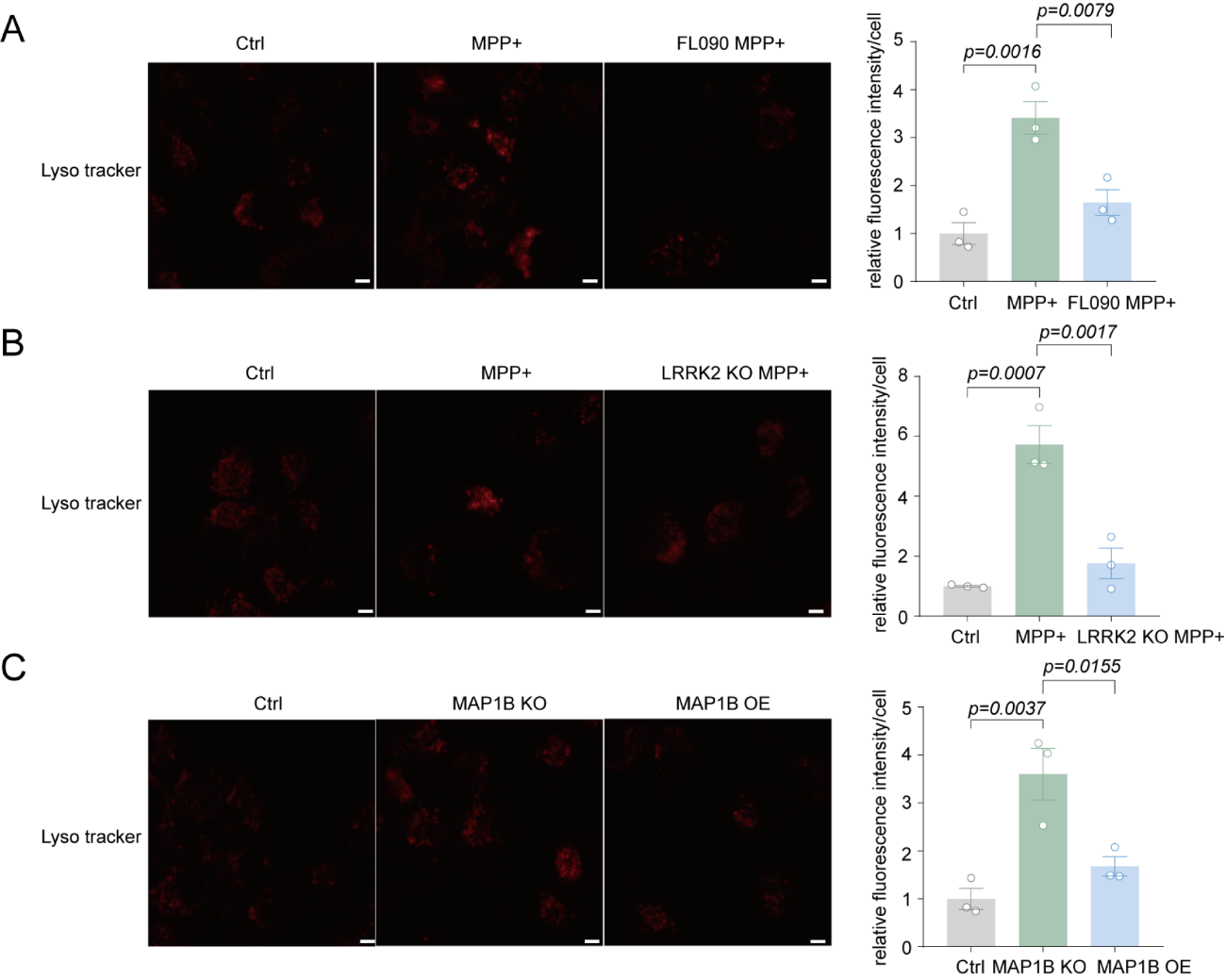
Supplementary Figure 5. **A** Staining and quantification of Lysosome tracker in the cells after 5 mM MPP+ and 1 μM FL090 co-treatment. MPP+-induced lysosomal damage was restored after FL090 treatment. Scale bar = 3 μm. Data are means ± SEM, n=3. One-way ANOVA with post-hoc Tukey test was performed. **B** Staining and quantification of Lysosome tracker in the WT and LRRK2 KO cells after 5 mM MPP+ treatment. MPP+-induced lysosomal damage was restored in LRRK2 KO cells. Scale bar = 3 μm. Data are means ± SEM, n=3. One-way ANOVA with post-hoc Tukey test was performed. **C** Staining and quantification of Lysosome tracker in the MAP1B knockout and MAP1B overexpression cells. Lysosomal damage occurred in MAP1B KO cells. Scale bar = 3 μm. Data are means ± SEM, n=3. One-way ANOVA with post-hoc Tukey test was performed. The p value as indicated in the figure. Each point on the graph represents a sample.


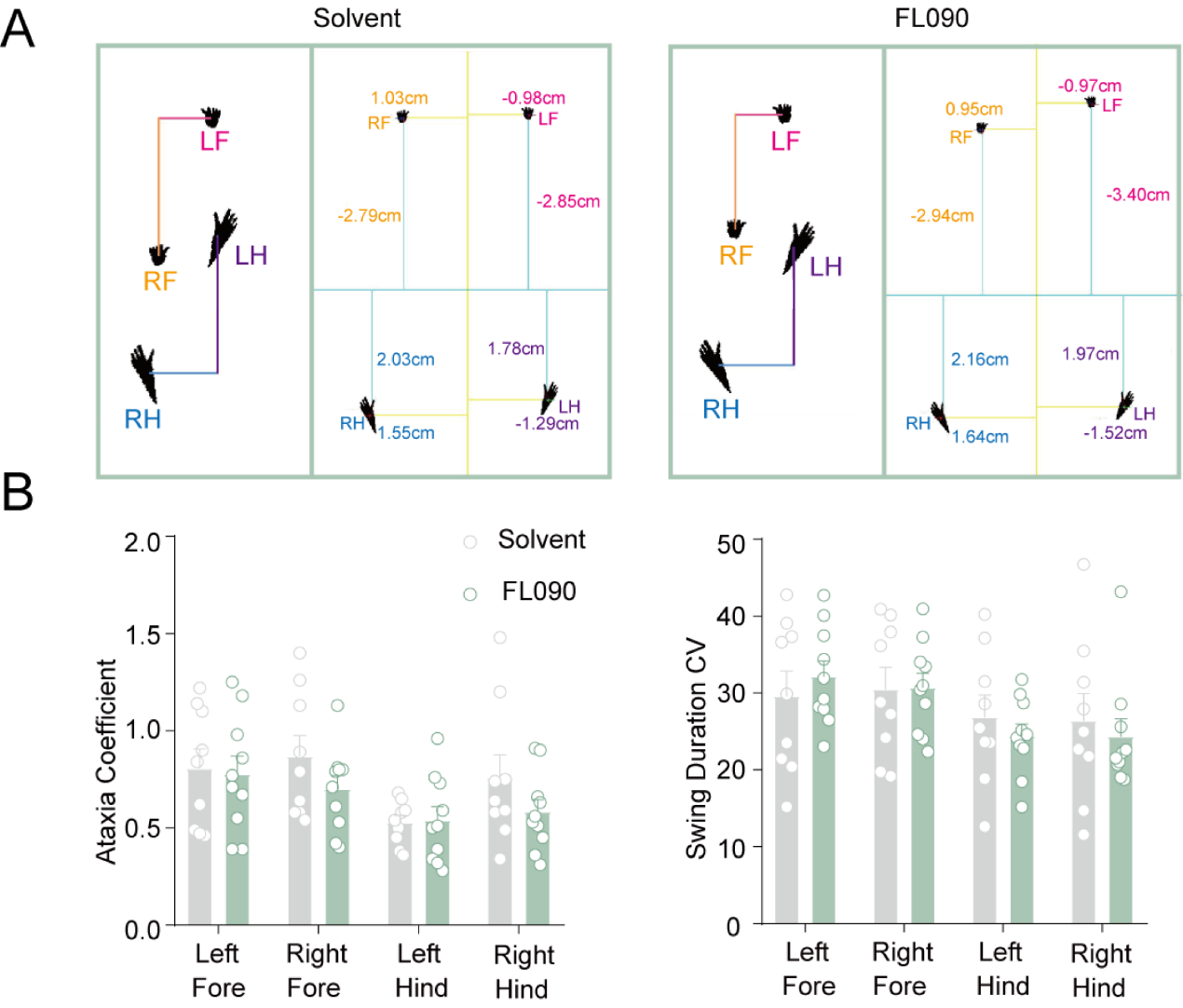


Supplementary Figure 6. FL090 treatment didn’t affect motor functions **A-B** The swing duration of CV and Ataxia Coefficient were obtained from mice in the Solvent, and FL090 groups. The value on the Y-axis represents the raw data exported from the DigiGaitTM analysis system. Data are means ± SEM. Solvent, n = 9; FL090, n=10. Two-way ANOVA with post-hoc Sidak test was performed. The p values lower than 0.05 are labeled in the histogram; otherwise, the p values are not displayed. Each point on the graph represents a sample.


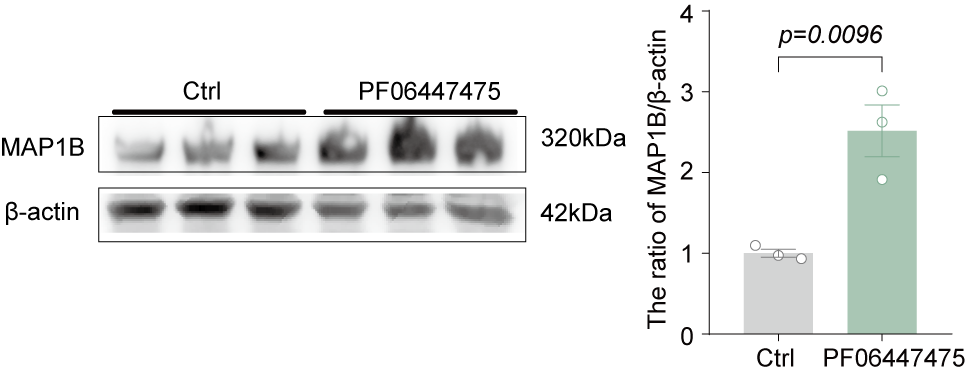


Supplementary Figure 7. MAP1B protein levels were examined in the N2a cells after 30 nM PF06447475 or DMSO treatment. Western blots were analyzed with Image J and normalized to β-actin expression. Data are means ± SEM, n = 3. t-test was performed. The p value as indicated in the figure. Each point on the graph represents a sample.


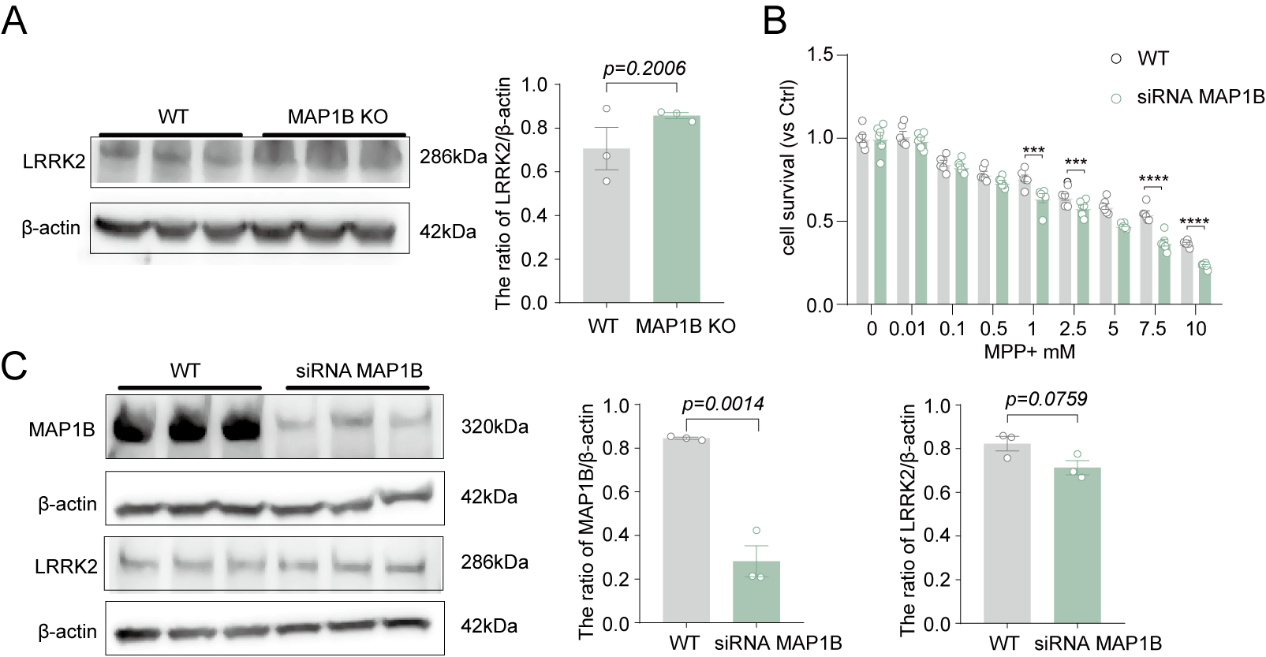
Supplementary Figure 8. Knockdown of MAP1B exacerbated cell death induced by MPP+. **A** LRRK2 protein level was examined in the knockout of MAP1B cells. Western blots were analyzed with Image J and normalized to β-actin expression. Data are means ± SEM, n = 3. t-test was performed. **B** Cell viability in WT cells and Knockdown MAP1B cells 24 h after MPP+ of concentration gradient treatment. Data are means ± SEM, n = 6 wells from one representative of 3 independent experiments. Two-way ANOVA with post-hoc Sidak test was performed. **C** LRRK2 and MAP1B protein levels were examined in the knockdown of MAP1B cells. Western blots were analyzed with Image J and normalized to β-actin expression. Data are means ± SEM, n = 3. t-test was performed. The p value as indicated in the figure. Each point on the graph represents a sample.

Figure 1 D, E, H


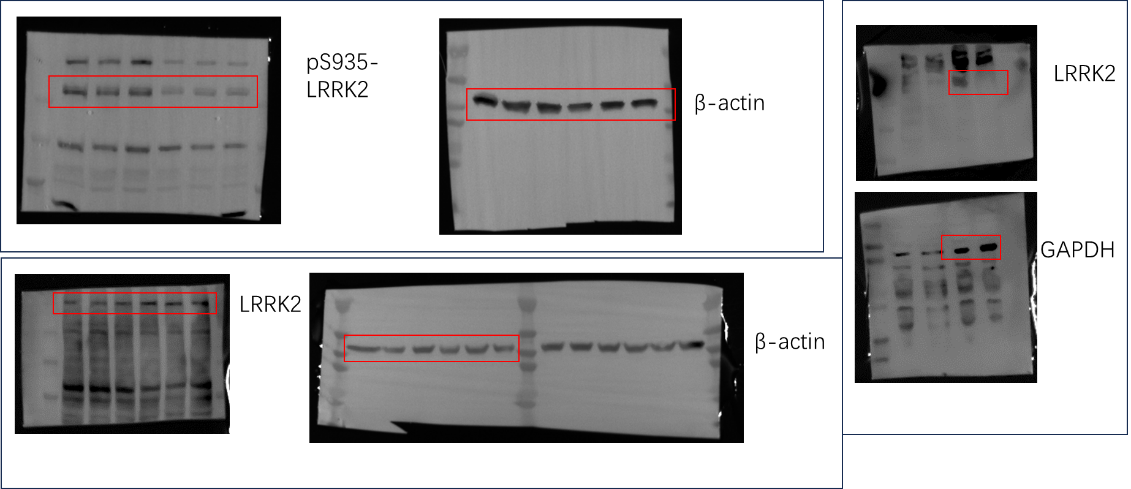


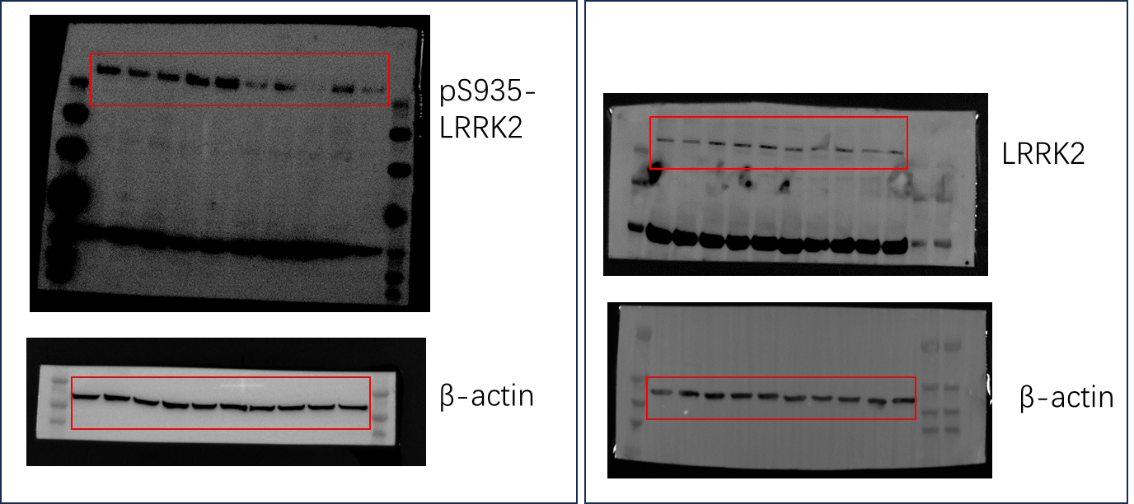


Figure 2E


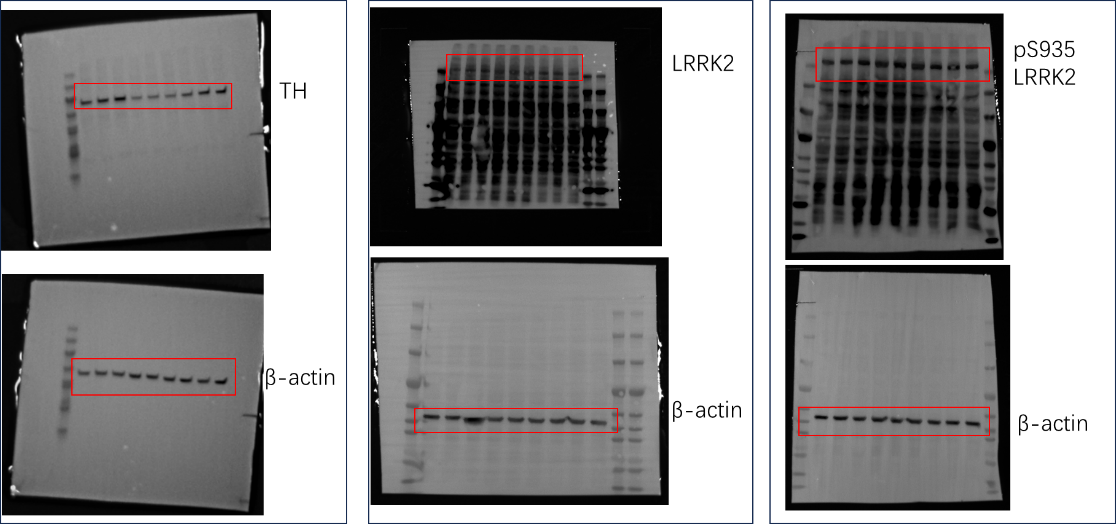


Figure 3C


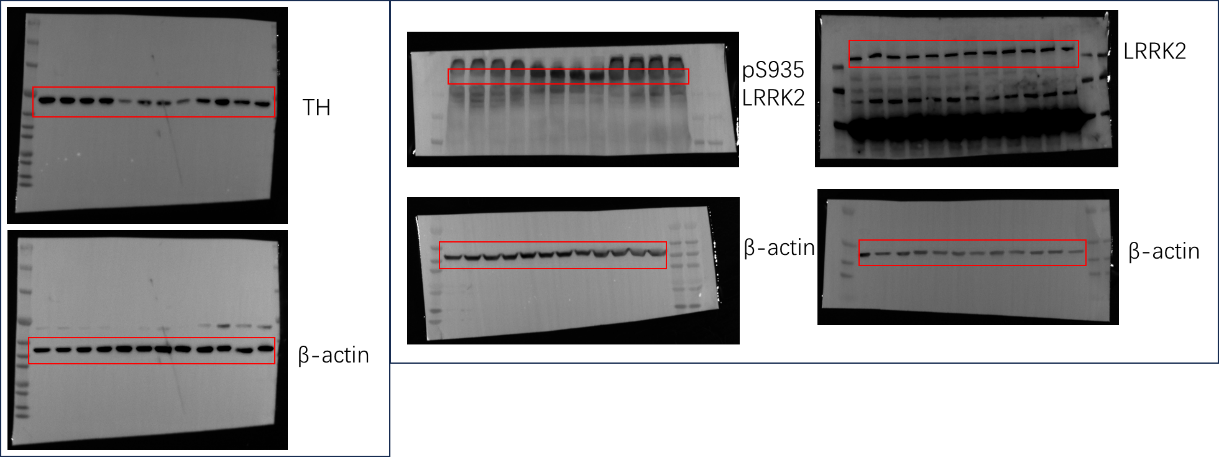


Figure 5 A, B, C


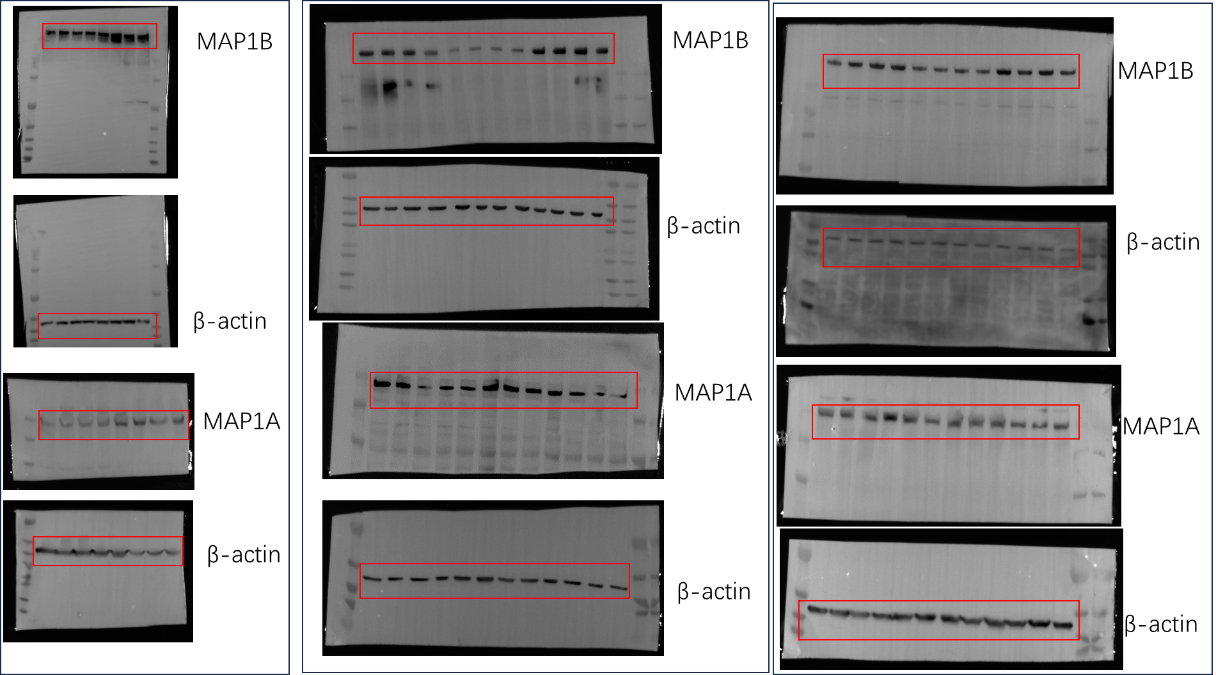


Figure 5E, F, G


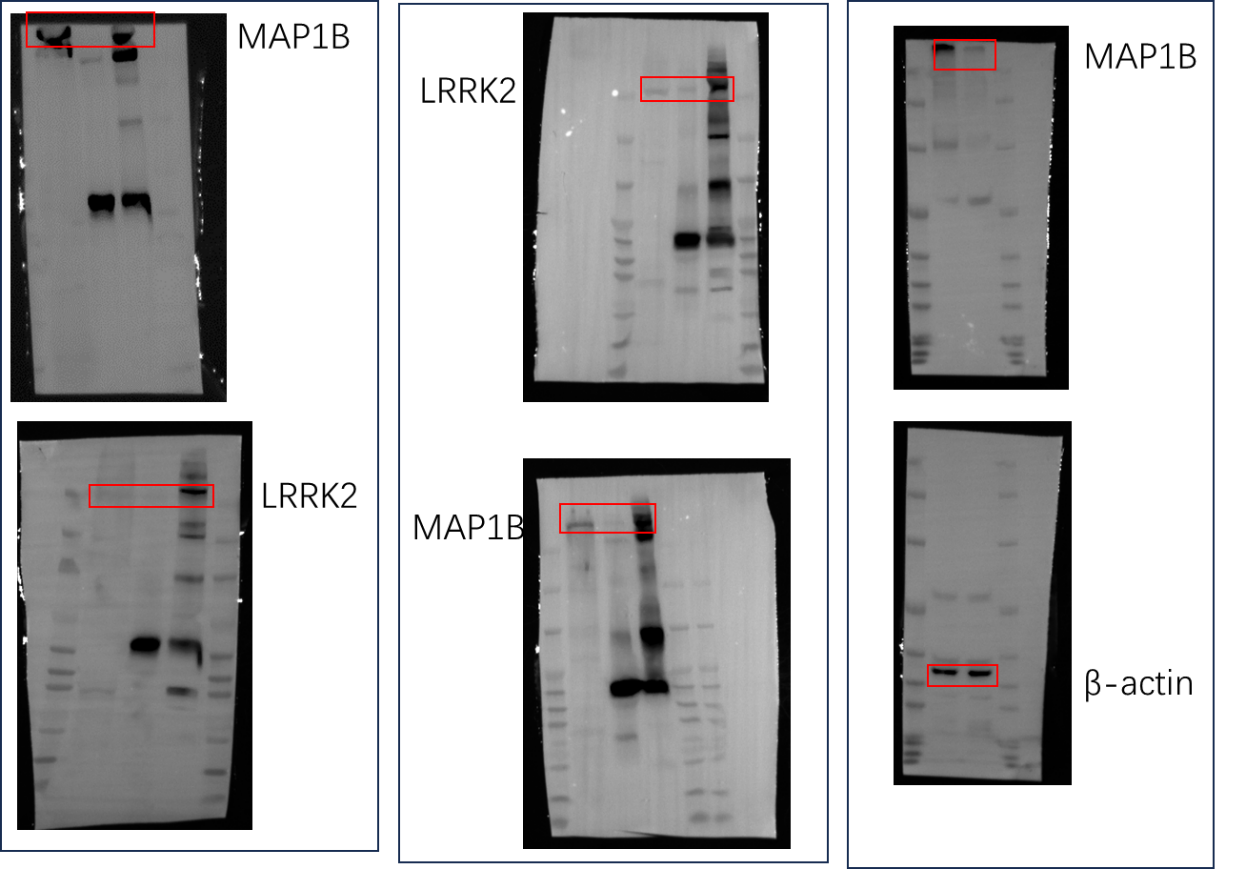


Figure 6 A, C, E


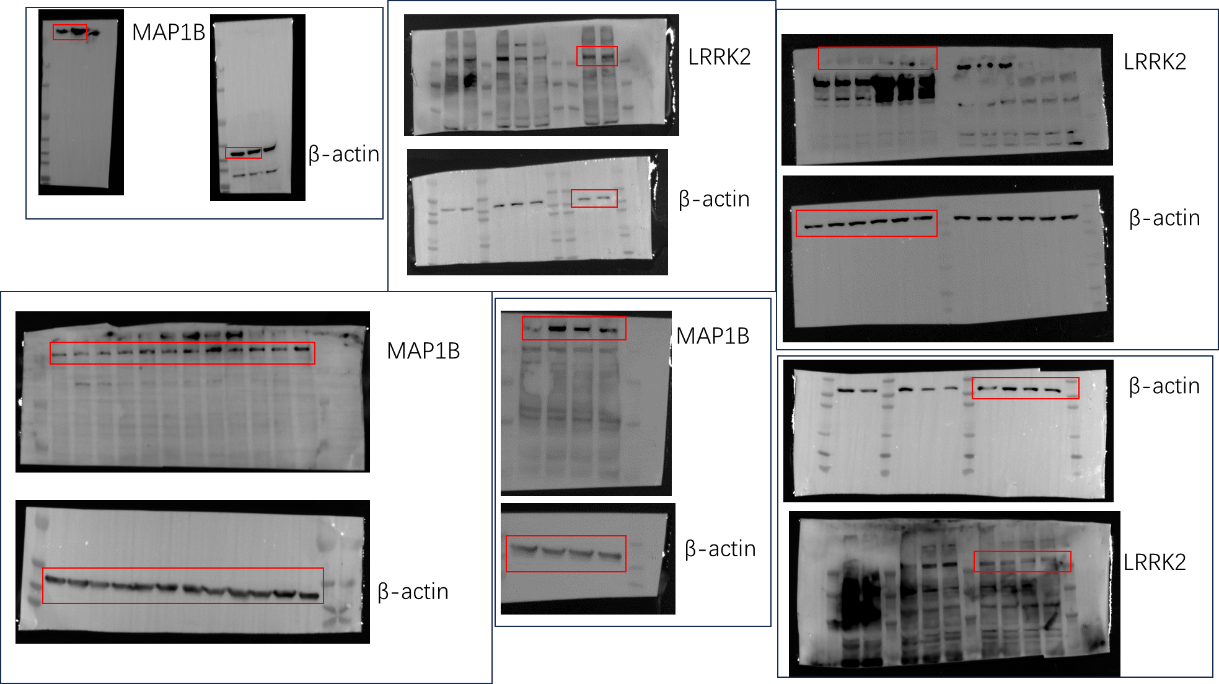


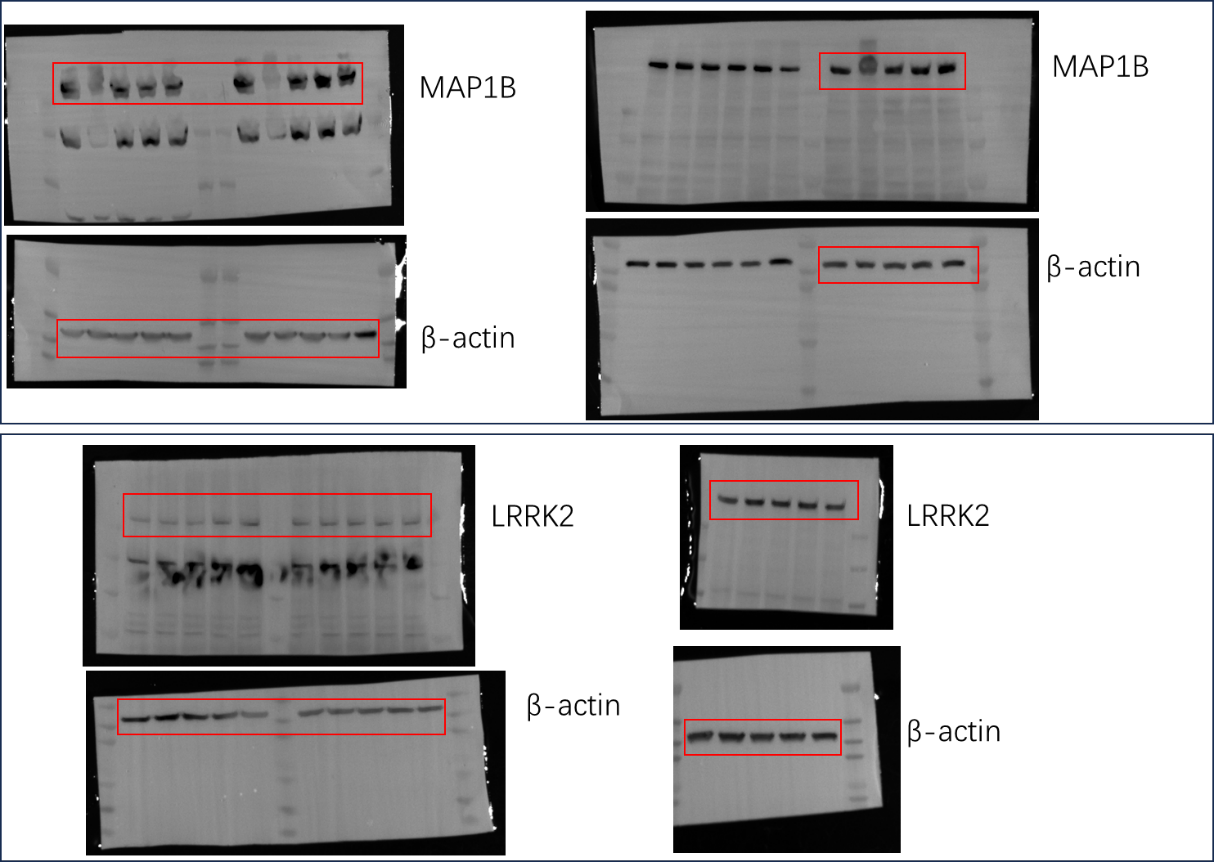


Figure S3


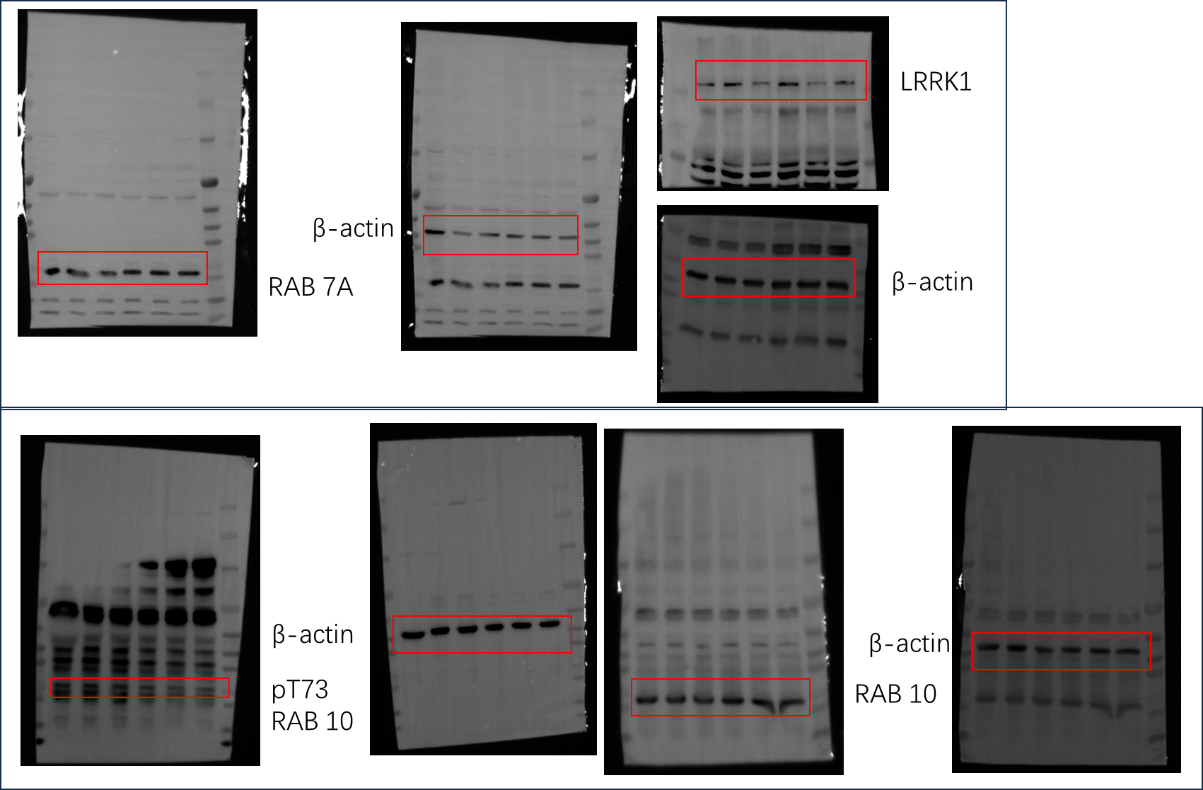


Figure S4A


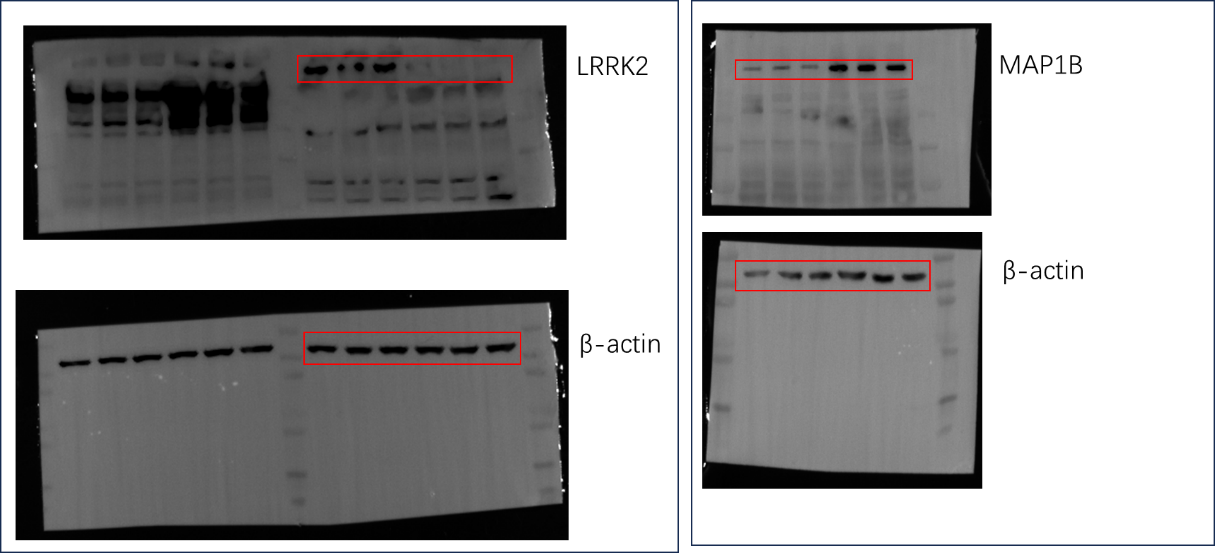


Figure S7


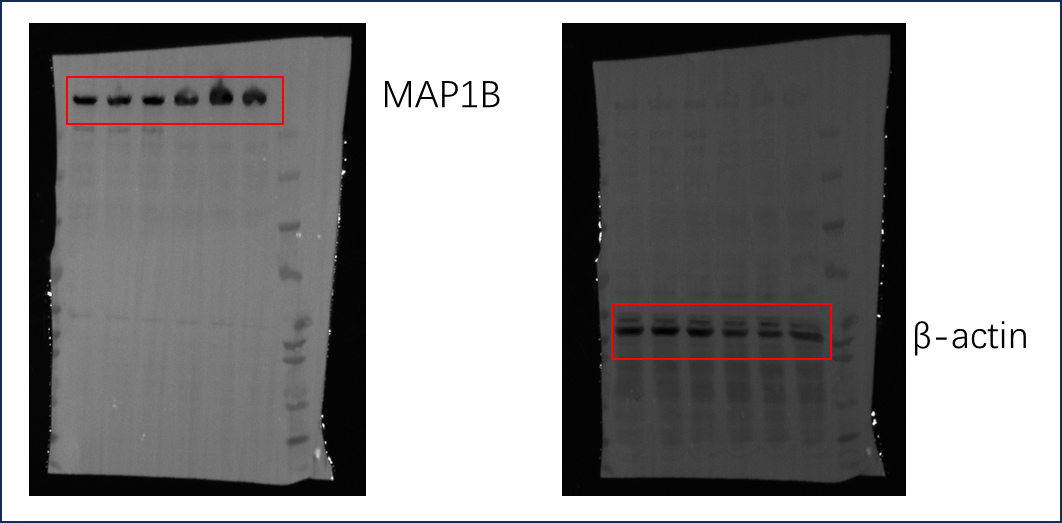


Figure S8A, C


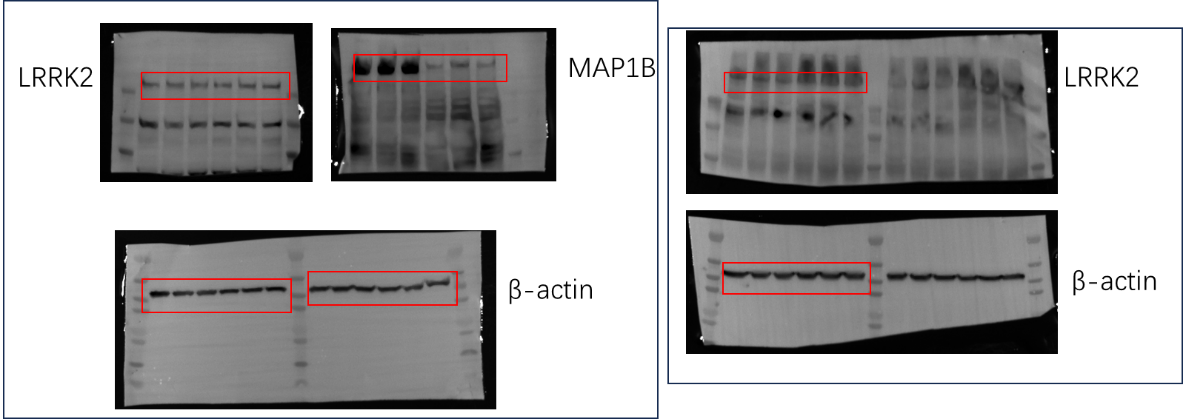


Supplementary Figure 9. Scans of uncropped blots. Red boxes highlight sections of blots shown in indicated figures.
